# Deciphering the Impact of AKT1 Pathogenic Variants in Juvenile Granulosa Cell Tumors Using a *Drosophila* Model

**DOI:** 10.1016/j.mcpro.2025.101466

**Published:** 2025-11-13

**Authors:** Reiner A. Veitia, Laetitia Herman, Bérangère Legois, Sandra Claret, Alain Zider, Anne-Laure Todeschini

**Affiliations:** 1 Université Paris Cité, CNRS, Institut Jacques Monod, Paris, France; 2 UniversitéParis Saclay, Saclay, France; 3 Institut de Biologie François Jacob, CEA, Fontenay-aux-Roses, France

**Keywords:** Juvenile granulosa cell tumors (GCTs), in-frame duplication, Drosophila model, RNA-Seq and mass spectrometry

## Abstract

Juvenile-type granulosa cell tumors (JGCTs) manifest during the prepubertal period as precocious pseudo-puberty and/or dysmenorrhea. We have previously identified pathogenic variants in AKT1 in JGCTs. This study aims to understand how these variants affect cellular function at the phenotypic and molecular levels using a *Drosophila* model. Transgenic *Drosophila* models expressing WT AKT1 and four pathogenic variants were created under the control of tissue-specific promoters. Phenotypic effects were studied by assessing *Drosophila* wings for cell division and growth using wing surface and trichome density and ovarian follicular cells were examined for subcellular localization and morphology. Molecular analyses included mass spectrometry to identify differentially expressed proteins (DEPs) and phospho-peptides, along with RNA-Seq to characterize transcriptomic changes. Wings expressing mutated AKT1 showed increased surface area and reduced trichome density, indicating larger cells. In ovarian follicular cells, WT AKT1 localized primarily to the cytoplasm, while mutated AKT1 variants were associated with the plasma membrane, leading to morphological abnormalities and increased cell size. Mass spectrometry revealed numerous DEPs and phospho-peptides, highlighting changes in pathways such as glycolysis and Rho GTPase signaling. Transcriptomics demonstrated a clear gain of function for mutated AKT1 in activating a subset of genes. However, several genes upregulated by WT AKT1 were less effectively activated by the mutants, indicating a potential loss-of-function in transcriptional regulation for this subset, revealing an unexpected mechanistic complexity. Network analysis of interactions involving DEPs, phosphorylated proteins, and transcription factors suggests these elements mediate the observed proteomic and transcriptional alterations. Taken together, the results underscore the utility of *Drosophila* models in unraveling the biological relevance of AKT1 pathogenic variants in cancer.

## Highlights

• Pathogenic AKT1 variants found in juvenile granulosa tumors were introduced into a *Drosophila* model.
• AKT1 variants cause abnormal cell growth, polarity defects and morphological alterations in tissues.
• Mass spectrometry revealed specific proteomic signatures associated with mutant AKT1 activity.
• Transcriptomics revealed unexpected gain-of-function and loss-of-function effects for distinct gene sets.
• Network analysis linking DEPs, phosphoproteins, and TFs, highlight AKT1 as key cancer driver.

## Graphical Abstract

**Figure:**
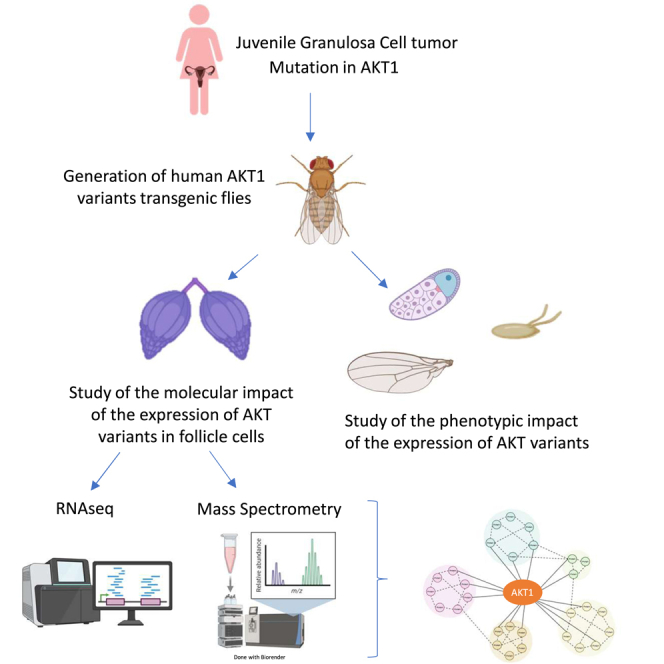

## In Brief

We use transgenic *Drosophila* to investigate how pathogenic AKT1 variants, identified in Juvenile granulosa cell tumors, impact cellular behavior. Combining *in vivo* phenotypic analysis with mass spectrometry and RNA-Seq, we reveal that AKT1 variants promote abnormal growth and polarity defects, accompanied by distinct proteomic and transcriptomic changes. These findings highlight the complexity of oncogenic AKT1 signaling and underscore the value of *Drosophila* models for dissecting tumorigenic mechanisms.

Sex cord stromal tumors involve granulosa, theca or stromal cells of the ovary. Ovarian granulosa cell tumors (GCTs) are the most common frequent ones and represent up to 8% of all ovarian tumors (1, 2). Five percent of GCTs occur in the prepubertal period. Such Juvenile-type GCTs (JGCTs) are often revealed by precocious pseudo-puberty and/or dysmenorrhea (3). Most JGCTs are detected at early stages but recurrence and advanced-stage tumors are possible (4). The study by Kalfa *et al*. reported the presence of activating somatic pathogenic variants of *Gαs* in one-third of the JGCTs analyzed, which might lead to a constitutive activation of mitogenic follicle-stimulating hormone receptor signaling (5). In previous studies, we searched for pathogenic variants in the genes coding for the serine/threonine protein kinases AKT1, AKT2, and AKT3 in a series of JGCTs negative for *GαS* pathogenic variants. We were able to show that more than 60% of the JGCT samples harbored in-frame duplications within *AKT1*, leading to its strong activation (6, 7). Single nucleotide variants in *AKT1* were also found scattered along the coding sequence in duplication-negative tumors. However, their status as drivers of tumorigenesis is not clear. Finally, a transcriptome-wide mutational analysis of four tumor samples failed to reveal any common pathogenic variants other the tandem duplications suggesting that the latter are major driver events in JGCT pathogenesis.

**Fig. 1:**
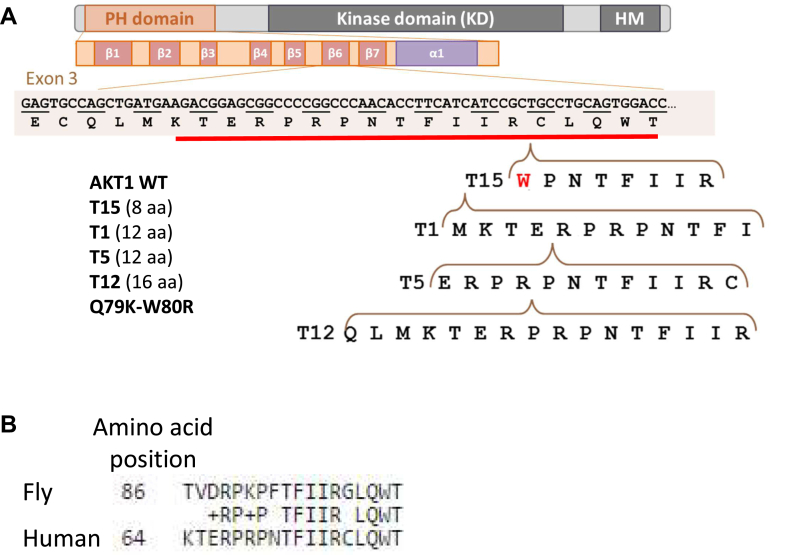
**Tandem duplications within exon three of AKT1 in JGCTs used in this study**. *A*, simplified representation of the AKT1 protein and of the PHD with its secondary structural features (beta strands from 1–7 and the C-terminal helix, according to crystallographic data from the Protein Data Bank). The sequences of four different duplications used in our study are displayed. The tandemly duplicated sequences are highlighted by *horizontal brackets* pointing to the position of the insertion in the WT sequence. T15 duplication breaks a codon, which leads to a change of one residue (in *red*). *B*, alignment of the human and fly AKT1 orthologous protein sequences. Only the region where the insertions occur is shown. JGCT, Juvenile-type granulosa cell tumor; PHD, pleckstrin homology domain.

AKT is a crucial effector of PI3K signaling. AKT proteins regulate cell survival, metabolism, migration, growth and proliferation, and other critical cellular functions. As outlined above, AKT activity results from the contribution of three paralogs in mammals AKT1-3. They have redundant and specific roles in mice. AKT proteins contain three main domains: the pleckstrin homology domain (PHD) located at the N terminus, a central kinase domain and a regulatory domain containing a hydrophobic motif at the C terminus. The tandem duplications we found in JGCTs affect the PHD of AKT1 (8). The PHD has two main beta sheets (Protein Data Bank structures: 1H10, 3O96, and 4EJN) and the tandem duplications affect the sixth beta strand (Fig. 1*A*). The PHD binds to phosphatidylinositol-diphosphate/triphosphate mainly localized at the plasma membrane. Transient relocalization of AKT1 to the membrane allows its phosphorylation by phosphoinositide-dependent protein kinase 1 on Thr308 and by mTOR complex 2 on Ser473. Full activation is achieved upon phosphorylation of S473 (9, 10). We also found two co-occurring substitutions Q79K and W80R located right after the beta strand affected by the duplications. Not surprisingly, Q79K activates AKT1 probably by decreasing the interaction between the PHD and the kinase domain (9, 11) and W80 is thought to interact with F469 from the hydrophobic domain to keep AKT in a closed/inactive conformation (12). The AKT1 proteins harboring the tandem duplications or the two point pathogenic variants were insensitive to serum deprivation, hyper-phosphorylated, and strongly localized at the plasma membrane in HeLa cells (6, 7).

Similar insertions have been described more recently in other cancer types such as breast cancer (13, 14, 15, 16) and in sclerosing pneumocytoma (17). In agreement with our previous results (6), Chang *et al*. 2017 showed that the expression of AKT1 P68_C77dup in MCF10A cells resulted in a higher level of AKT phosphorylation at residues Thr308 and Ser473 and to a stronger increase of the phosphorylation of AKT1 downstream effectors such as S6 and PRAS40, than the two most common AKT1 missense variants, E17K and Q79K. Moreover, isogenic cells expressing mutated AKT1 (p.68_C77dup) displayed a greater sensitivity to the ATP-competitive pan-AKT inhibitor AZD5363 than cells expressing AKT1E17K or the WT counterpart (16).

AKT is a well-studied kinase and many downstream targets have been reported. For instance, AKT promotes cell survival via the phosphorylation of BCL2-associated agonist of cell death or the activation of mouse double minute 2 homolog. In addition, it modulates the activity of several transcription factors (TFs) (18). For instance, AKT regulates cell survival by inhibiting the phosphorylation of Forkhead box O factors, which leads to their nuclear export or by activating CAMP responsive element-binding protein 1 and NFκB. AKT stimulates cell division by inhibiting cell-cycle inhibitors, such as p27 or by phosphorylating glycogen synthase inase 3 (GSK3). GSK3 inhibition also regulates nutrient uptake at the plasma membrane and promotes energy storage. AKT also promotes cell growth by regulating the mammalian target of rapamycin (mTOR) complex 1, which regulates translation initiation and ribosome biogenesis. It also promotes cell migration and invasion by regulating actin cytoskeleton dynamics and the production of matrix metalloproteases ((19) and references therein).

As discussed previously, the insertions in AKT1 were the sole detectable lesions common to the JGCTs we studied, which strongly suggest that they are the driving event in these tumors. However, little is known about their effects at the molecular level. Thus, in this study, we have performed high-throughput analyses of the proteins and transcripts deregulated after mild overexpression of human WT and mutated *AKT1* variants in *Drosophila*, which is a powerful yet-simpler-than-mouse model for studying cancer (20). We found that WT and mutated AKT1 had distinct subcellular localizations and effects on cell and tissue morphology. Mutated AKT1 variants were found to cause follicle cell enlargement, induced abnormal tissue morphogenesis like abnormal dorsal appendages, and led to the presence of larger cells in the wings as evidenced by an increased surface area along with a reduced trichome density. At the transcriptional level, mutated AKT1 showed both gain-of-function effects for some genes and potential loss of function for others, indicating complex regulatory impacts. The analysis of mass spectrometry (MS) data pointed to several perturbed pathways and networks. Overall, our findings demonstrate the value of *Drosophila* models in understanding the biological effects of AKT pathogenic variants in the context of human cancer.

## Experimental Procedures

### Fly Stock

The WT control stock used throughout this work was the 51C fly line, named LP (for landing pad) hereafter. The *tj-GAL4* line was provided by the Kyoto *Drosophila* stock center, the *ptc-GAL4*, the *en-GAL4*, and the *gmr-GAL4* strains came from the Bloomington *Drosophila* center. All fly lines were cultured on standard cornmeal media at 18 °C, 22 °C, or 25 °C. The studies were approved by the Ministère de l’enseignement supérieur, de la recherche et de l’innovation.

### Transgenes

The various AKT1 forms were amplified from plasmids described in Bessière *et al* 2015. EcoR1–AKT1-Xho1 fragments were generated in one-step PCR and cloned (EcoR1–Xho1) into digested pUASTattb vector (DGRC Stock 1419; https://dgrc.bio.indiana.edu//stock/1419; RRID:DGRC_1419) (21). All constructs were sequenced to exclude the presence of PCR-induced mutations. The sequences of the primers used are the following:

### XhoI-3XFLAG-AKT-R

atgcatctcgagCTACTTGTCATCGTCATCCTTGTAGTCGATGTCATGATCTTTATAATCACCGTCATGGTCTTTGTAGTCGGCCGTGCCGCTGGCCGAGTAGGAGAAC

EcoRI-AKT-F

atgcatgaattcATGAGCGACGTGGCTATTGTGAAGG

### Generation of UAS-AKT1 Transgenic Flies

Site-specific insertion of pUASTattB-AKT1-3xFLAG constructs into the 51C fly line (y1 M{vas-int.Dm}ZH-2A w∗; M{3xP3-RFP.attP'}ZH-51C) (Reference BDSC Stock #24482) was performed by BestGene, Inc. Viable lines were maintained as balanced stocks by conventional crosses. Confirmation of the presence of the correct AKT1 transgene at the 51C site was carried out by PCR of genomic DNA followed by Sanger sequencing. Briefly, one fly was placed into a tube, homogenized for 10 s in 50 μl of squishing buffer (10 mM Tris-Cl pH = 8.2, 1 Mm EDTA, 25 mM NaCl, and 200 μg/ml proteinase K) and incubated at 37 °C for 30 min. Proteinase K was inactivated by heating to 95 °C for 5 min. One microliter of homogenate was used to perform PCR analysis.

### Western Blotting of Adult Fly Ovaries and Antibodies

Six fly ovaries were homogenized in ice-cold radioimmunoprecipitation assay buffer (50 mM Tris–HCl, pH 8.0, 150 mM sodium chloride, 1.0% NP-40, 0.5% sodium deoxycholate, 0.1% sodium dodecyl sulfate, 1 × protease inhibitor Complete Mini EDTA-free, and 1 × PhosSTOP (both from Roche) and quickly placed on ice. The samples were then centrifuged at 13,000 rpm for 10 min and the supernatant transferred to a new tube. Then, 4 × lithium dodecyl sulfate sample buffer (Life Technologies Corporation) was added before boiling the samples for 5 min. SDS-PAGE and Western blot were performed according to standard procedures (NuPAGE Novex 10% Bis-Tris Gel 1.0 mm, iBlot 2 Dry Blotting System, both from Life Technologies Corporation). The antibodies used were (reference, dilution): phospho-AKT (S473) (sc-7985-R, 1:1000), total AKT (sc-8312, 1:1000), octA (3xFLAG, sc-166384, 1:1000), Tubulin (YL1/2, sc-53029, 1:5000) (all from Santa Cruz Biotechnology), phospho-AKT (Thr450) (Assay Biotech A0406, 1:500), and anti-GAPDH (ABM G041, 1:1000, Applied Biological Materials, Inc). Antibodies were diluted in 5% milk PBS with 0.1% tween. Membranes were washed 3 times for 10 min and incubated with a 1:20,000 dilution of horseradish peroxidase–conjugated anti-rabbit (Jackson ImmunoResearch Laboratories; #111-035-003) or anti-mouse (Jackson ImmunoResearch Laboratories, # 115-035-003) antibodies for 1 h 30 min. After this step, blots were washed 3 times with PBS + 0.1% tween and detection was performed with the SuperSignal West Pico Chemiluminescent Substrate (Pierce) according to the manufacturer’s protocol. Image acquisition was performed using the ImageQuant LAS4000 software on the LAS4000 device (https://www.gehealthcare.fr/about/life-sciences, GE Healthcare).

### Immunostaining and Imaging

Immunostaining was performed using standard protocols (22). Antibody dilutions were as follows: rabbit anti-phosphoAKT (S473) (sc-7985-R, 1:1000); rabbit anti-AKT (sc-8312, 1:1000); mouse anti-octA (sc-166384, 1:1000), all from Santa Cruz Biotechnology. F-Actin was stained with rhodamine-phalloidin (1:200; Molecular Probes). Membrane glycoproteins were stained with rhodamine-wheat germ agglutinin (1:100; Molecular Probes). Hoechst 33342 was used to stain DNA (Life Technologies Corporation,). Images were obtained with a LSM 710 (Zeiss) microscope.

### Preparation of Follicle Cells

Preparation of follicle cells was adapted from Bryant *et al*. PNAS 1999 (23). Ovaries were dissected in Schneider’s S2 insect medium, supplemented with 10% fetal calf serum. Ovaries (30 pairs) were washed three times in calcium-free PBS and incubated at 22 °C for 30 min with vigorous shaking (2000 rpm) in 1 ml of 0.05% trypsin and 5 mg/ml collagenase in PBS. After the removal of the supernatant the remaining cell/tissue material was filtered through a 30 μM nylon mesh and collected in tubes containing 1 ml of S2 medium, pelleted at 1000*g* for 5 min in a microcentrifuge and washed in 1 ml of PBS. For RNA-Seq experiments, the pellets were resuspended directly in TRI-reagent (Molecular Research Center) and store at −80 °C. For MS, the pellets were resuspended directly in radioimmunoprecipitation assay buffer and stored at −80 °C.

### RNA Extraction and Sequencing

RNA extraction was performed using the TRI-reagent supplier’s instructions (Molecular Research Center). Total RNA quality was assessed on an Agilent Bioanalyzer 2100, using the RNA 6000 pico kit (Agilent Technologies). One microgram of total RNAs underwent high-throughput sequencing at the Genom’IC platform (from Cochin Institute).

### Quantitative Reverse Transcription Polymerase Chain Reaction

Transgene expression and the quality of follicle cell purification were evaluated by quantitative reverse transcription polymerase chain reaction (RT-qPCR). RNA extraction was performed using the TRI-reagent following supplier’s instructions. Complementary DNA synthesis was performed with 1 μg of total RNA after DNase treatment (New England BioLabs), using the M-MLV RT enzyme according to the manufacturer’s protocol (Thermo Fisher Scientific). RT-qPCR was performed using the GoTaq qPCR Master Mix 5X (Promega) in the Stratagene Mx3000P qPCR System.

### Examination of the Wings

Adult wings were dissected in 95% ethanol and transferred to a drop of Euparal mounting medium (Bioquip.com) on a clean slide (Thermo Fisher Scientific). The slide was overlaid by a clean microscope cover slip (Thermo Fisher Scientific) and after adding Euparal (Chroma). Slides were baked overnight at 37 °C to slightly harden the Euparal. Wing pictures were taken with a Keyence VHX-2000 Z20 microscope (x20 or x100). Contours of wing areas were manually defined using ImageJ (https://imagej.net/software/fiji/downloads, 1.50 d, java 1.8.0_212, 64 bit) (n = 20 per genotype except n = 9 for Q79KW80R) (24). To determine cell density a rectangular region of interest (ROI) was selected and a ROI of the same surface was used for all genotypes. Trichome were then manually counted to determine the average number of cells per ROI (n = 10 per genotype except n = 9 for Q79KW80R). We used the same pictures to measure the orientation of each trichome in the region expressing the transgene using ImageJ’s “directionality” plug-in (25).

### MS Analysis

LC-MS/MS acquisition: Protein extracts (30 μg) from follicle cells were precipitated with acetone at −20 °C during 3 h and incubated with 20 μl of 25 mM NH4HCO3 containing sequencing-grade trypsin (12.5 μg/ml; Promega) overnight at 37 °C. The resulting peptides were desalted using ZipTip μ-C18 Pipette Tips (Pierce Biotechnology).

Samples were analyzed using either an Orbitrap Fusion (cells expressing WT or LP control) or an Orbitrap Q-Exactive Plus (cells expressing WT and mutant forms), coupled respectively to a Nano-LC Proxeon 1200 or a Nano-LC Proxeon 1000, both equipped with an easy spray ion source (Thermo Fisher Scientific). On the Orbitrap Fusion instrument, peptides were loaded with an online preconcentration method and separated by chromatography using a Pepmap-RSLC C18 column (0.75 × 750 mm, 2 μm, 100 Å) from Thermo Fisher Scientific, equilibrated at 50 °C, and operated at a flow rate of 300 nl/min. Peptides were eluted by a gradient of solvent A (H2O, 0.1% formic acid (FA) and solvent B (acetonitril/H2O 80/20, 0.1% FA), the column was first equilibrated 5 min with 95% of A, then B was raised to 28% in 105 min and to 40% in 15 min. Finally, the column was washed with 95% B during 20 min and re-equilibrated at 95% A during 10 min. Peptides masses were analyzed in the Orbitrap cell in full ion scan mode, at a resolution of 120,000, a mass range of *m/z* 350 to 1550 and an automatic gain control (AGC) target of 4.105. Tandem mass spectrometry (MS/MS) were performed in the top speed 3 s mode. Peptides were selected for fragmentation by higher-energy C-trap dissociation with a normalized collisional energy of 27% and a dynamic exclusion of 60 s. Fragment masses were measured in an ion trap in the rapid mode, with and an AGC target of 1.104. Monocharged peptides and unassigned charge states were excluded from the MS/MS acquisition. The maximum ion accumulation times were set to 100 ms for MS and 35 ms for MS/MS acquisitions respectively.

On the Q-Exactive Plus instrument, peptides were loaded with an online preconcentration method and separated by chromatography using a Pepmap-RSLC C18 column (0.75 × 500 mm, 2 μm, 100 Å) from Thermo Fisher Scientific, equilibrated at 50 °C and operated at a flow rate of 300 nl/min. Peptides were eluted by a gradient of solvent A (H2O, 0.1% FA) and solvent B (100% acetonitril, 0.1% FA), the column was first equilibrated 5 min with 95% of A, then B was raised to 35% in 93 min and finally, and the column was washed with 80% B during 10 min and re-equilibrated at 95% A during 10 min. Peptides masses were analyzed in the Orbitrap cell in full ion scan mode at a resolution of 70,000 with a mass range of *m/z* 375 to 1500 and an AGC target of 3.106. MS/MS were performed in a Top 20 data dependent acquisition mode. Peptides were selected for fragmentation by higher-energy C-trap dissociation with a normalized collisional energy of 27% and a dynamic exclusion of 30 s. Fragment masses were measured in the Orbitrap cell at a resolution of 17,500, with an AGC target of 2.105. Monocharged peptides and unassigned charge states were excluded from the MS/MS acquisition. The maximum ion accumulation times were set to 50 ms for MS and 45 ms for MS/MS acquisitions, respectively.

### *In Silico* Analyses

To study the cellular processes differentially affected by WT and mutated *Akt1*, we used String Interactor v11 (https://string-db.org/) to build networks based on the physical interactions known to occur among the DEProts and DPhospho peptides (26). Enrichment analyses were performed with String Interactor v11 and Enrichr (for Human https://amp.pharm.mssm.edu/Enrichr/and for fly https://maayanlab.cloud/FlyEnrichr/). We used Cytoscape 3.8.0 to draw and display the networks (27).

### Overrepresentation Analysis and Bubble Plots

Overrepresentation analysis (ORA) was performed using PAthway, Network and Gene set Enrichment Analysis (https://www.flyrnai.org/tools/pangea/web/home/7227). The full ORA results are provided in relevant Additional tables. The enriched pathways highlighted in the text and/or figures were selected as follows: i) pathways were sorted based on their adjusted *p* values, ii) when pathways/terms from different databases (*e*.*g*.*,* gene ontology biological process, BioPlanet, etc.) occurred several times, the one with the smallest adjusted *p* value was kept, and iii) when related terms involving the similar gene subsets were present, only the most general term/pathway was retained. Terms/pathways not linked with ovarian function were not retained for the bubble plots.

### Experimental Design and Statistical Rationale

For RNA-Seq and MS, total RNA/protein from follicle cells were provided to the corresponding platforms (see previous sections). Specifically, we analyzed three control samples (LP), along with three samples for RNA-Seq and four samples for MS of WT AKT1 and three samples per mutant (4 AKT variants with tandem duplications and the Q79K-W80R double mutant). Each sample results from follicle cells purified from 30 pairs of ovaries stemming from individual crosses (*i*.*e*.*,* biological replicates, see Preparation of follicle cells section).

Differential mRNA expression analysis was performed with DESeq2 (https://bioconductor.org/packages/devel/bioc/vignettes/DESeq2/inst/doc/DESeq2.html), comparing the three control LP samples against the 3 WT samples or comparing the 3 WT samples with the 12 “mutant” samples. The *p* values were adjusted for multiple testing using the Benjamini and Hochberg method, and those with an adjusted *p* value <0.05 were retained for further analyses. Data will be deposited in Gene Expression Omnibus during the peer-review period.

Peptide and protein identification and quantification: Label-free relative quantification was performed in between subject analysis using Progenesis-Qi software 4.0 (https://www.nonlinear.com/progenesis/qi-for-proteomics/v4.0/, Nonlinear Dynamics Ltd). For the identification step, all MS and MS/MS data were processed with the Proteome Discoverer software (https://www.thermofisher.com/fr/fr/home.html, Thermo Fisher Scientific, version 2.1) coupled to the Mascot search engine (Matrix Science, version 2.5.1). The database used contained 48507 entries. The mass tolerance was set to 6 ppm for precursor ions and 0.02 Da for fragments with the Q-exactive plus and 0.5 Da with the Fusion. The maximum number of missed cleavages was limited to two for the trypsin protease. The following variable modifications were allowed: oxidation (Met), phosphorylation (Ser, Thr, Tyr), and acetylation (Protein N-term). The SwissProt database (2017) with the *Drosophila melanogaster* taxonomy was used for the MS/MS identification step.

Peptide identification was validated using a 1% (false discovery rate) threshold calculated using the Percolator algorithm. The ptmRS node from Proteome Discoverer 2.1 was used to provide a confidence score of phosphorylated positions for each peptide. We kept those with a ptmRS binomial peptide score >50 (28). Protein abundance variations were measured according to the Hi-3 label-free quantification method and validated if their ANOVA *p* values were under 0.05. Proteomics data have been deposited to the ProteomeXchange Consortium via the PRIDE (29) partner repository with the dataset identifier PXD060549.

Although no phospho-peptide enrichment step was involved in our experiments, MS also detected phosphorylated peptides. We used this information to identify potential differentially phosphorylated proteins (DPhospho-peptides) between the WT and each of the mutated conditions. The abundance of the phosphopeptides was normalized to the corresponding protein levels from the total proteome. To identify the DPhospho-peptides, we used to strategies. For the first one, we proceeded as previously described by us (30). In short, we plotted the values of log_2_fold change (Log2FC) Expression *versus* the values of Log2FC Phosphorylation. To correct the intercept and make it = 0, we subtracted the offset of the trend line to the log2FC phosphorylation values. We then calculated the normalized differences between Log2FC expression (A) and corrected Log2FC phosphorylation (B) for each DPhospho peptides identified ([max(A.B) − min(A.B)]/(A + B)). Then, we detected the DPhospho peptides as the outliers according to an Iglewicz and Hoaglin's test (Z score ≥1, http://contchart.com/outliers.aspx). For the second one, to produce the volcano plot, we computed all possible ratios of the normalized abundance of each phosphopeptide in each mutant condition over the abundance of the same phosphopeptide in the control (WT) condition (*i*.*e*.*,* FC_Phospho_). A similar calculation was performed for the abundance of the relevant proteins (FC_Tot_). We added +1 to each value to avoid an “error” in the next step. We took the log2 of each value and calculated the average value for the phosphopeptides detected in each mutated condition (log2FC_Phospho_) and in the relevant control (log2FC_Tot_). Next, we calculated (log2FC_Phospho_) − (log2FC_Tot_) for each phosphopeptide present in each mutant. In parallel, we performed a Student’s *t* test between the log2FC_Phospho_ and the log2FC_Tot_ for each phosphopeptide in each mutant and applied a Bonferroni correction. The volcano plot was obtained the adjusted *p* values versus (log2FC_Phospho_) − (log2FC_Tot_). Data were plotted using R.

## Results

### Mutated Human AKT1 Variants Expression in *Drosophila* Cause Morphological Defects, Cell Growth, and Polarity Abnormalities

**Fig. 2:**
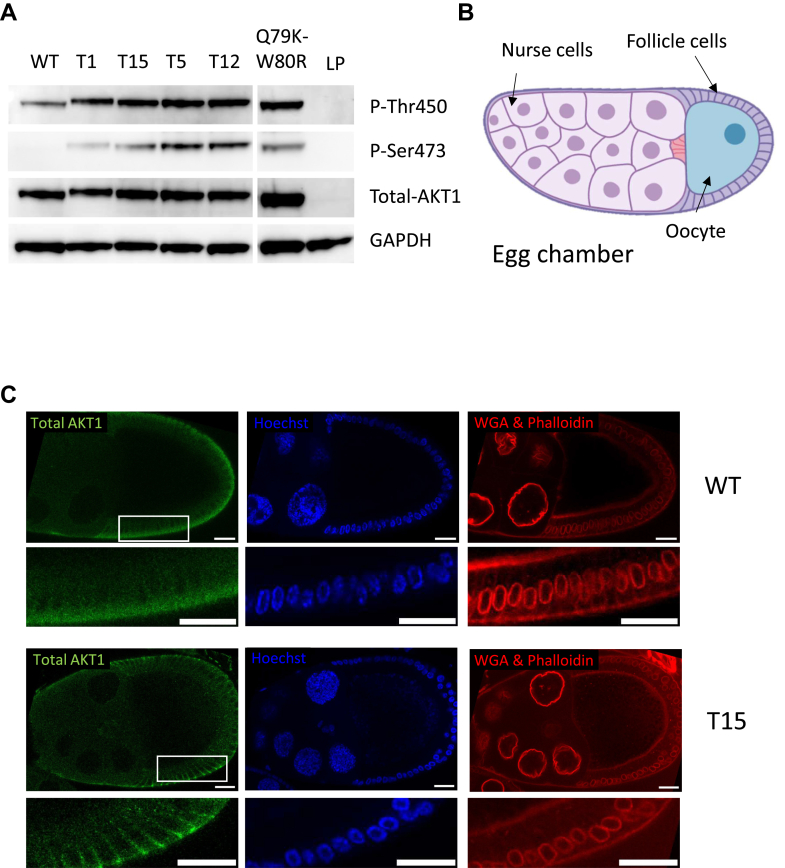
**Expression of WT and mutated forms of human AKT1 in drosophila follicular cells**. *A*, Western blot analysis of the total and phosphorylated AKT1 variants (WT, T1, T15, T5, T12, and Q79K-W80R). The LP line serves as a reference (no transgene). Protein extracts from the ovary of *Drosophila* females of the genotypes: *tj*-GAL4/landing pad (LP, no AKT1 expression), *tj*-GAL4/UAS-AKT1-WT, -AKT1-T1, -AKT1-T15, -AKT1-T5, -AKT1-T12, or -AKT1-Q79K-W80R were probed. The anti-GAPDH shows that similar protein amounts were loaded in each lane, showing that each line expressed a similar amount of AKT1. The anti-phospho Ser/Thr antibodies show a much stronger signal for the mutated proteins than for the WT. *B*, structure of an egg chamber, which consists of an oocyte (future egg) surrounded by somatic cells (follicle and nurse cells). *C*, immunostaining of egg chambers expressing either WT or T15 mutant AKT1 under the control of the driver *Traffic Jam*-GAL4. Actin was stained with phalloidin (in *red*), the plasma membrane by wheat germ agglutinin (in *red*), AKT1 by immunostaining (in *green*, anti-total AKT1), and nuclei are stained with Hoechst. The *white rectangles* indicate the positions that are zoomed in on the panels below each relevant panel to show the localization of WT or mutated AKT1 and the mislocalization of the nuclei in the mutants. Scale bars represent 20 μm.

*Drosophila* has been shown to be a powerful tool as a model for studying cancer (20). Here, we use this model organism to assess the phenotypic and molecular impact of the expression of some of the AKT variants that we have previously found in human JGCTs. The suitability of *Drosophila* is all the more relevant because the region where insertions occur is conserved between dAkt and AKT1 (Fig. 1*B*). Specifically, we generated transgenic lines allowing the expression of WT AKT, four AKT variants with tandem duplications and the Q79K-W80R double mutant (Fig. 1*A*). First, we expressed WT AKT and mutated AKT in ovarian follicle cells using the follicle-specific *traffic jam*-Gal4 driver (*tj*-GAL4). An immunoblot performed on ovarian cell extracts using polyclonal antibodies against human AKT (sc-8312) showed the expression of all of the variants of the oncoprotein (Fig. 2*A*). Additionally, an antibody directed against activated AKT, phosphorylated ser473, showed a clear hyperphosphorylation (and, hence, activation) of the mutated AKT variants (Fig. 2*A*).

To study the subcellular localization of the different human AKT1 variants in *Drosophila*, we performed an immunostaining in the ovary, using polyclonal antibodies against human AKT, and observed that WT AKT was mainly cytoplasmic in columnar follicle cells, whereas the mutated variants localized to the membrane (Fig. 2*C*). No signal was detected when immunostaining was performed with LP (CTRL) *Drosophila* strains (data not shown). The same experiment carried out with an antibody directed against the phospho-AKT1 (S473) clearly showed a membrane localization of mutated AKT1 (Supplemental Fig. S1). In addition, we consistently observed an abnormal follicle morphology when mutated AKT1 were expressed. Indeed, follicle cells expressing the mutated variants appeared abnormally enlarged. This was especially so for Q79K-W80R. This phenotype is reminiscent of the abnormally large follicles found in JGCTs. Interestingly, we also observed that nuclei localized closer to the basal membrane when mutated AKT variants were expressed, suggesting an impact of these AKT1 forms on apico-basal polarity or on other planar polarity issues (Figs. 2*C* and Supplemental Fig. S1).

We also screened the dorsal appendages for the presence of abnormalities (Supplemental Fig. S2). These structures correspond to breathing tubes that are formed by groups of dorsal follicle cells. Their formation is controlled by signaling cascades involving bone morphogenetic protein, epidermal growth factor and Notch (31). We observed a significant proportion of shorter and/or abnormal respiratory appendages in the embryos expressing mutated AKT1 (Supplemental Fig. S2*A*). The UAS-GAL4 system is temperature-dependent: a higher temperature enhances the activity of the GAL4 protein, leading to stronger expression of the UAS-linked gene. As shown in Supplemental Figure S2, *B* and *C*, the number of embryos carrying abnormal appendages varied from one mutant to another with an increase in number with the increase of the temperature from 22 °C to 25 °C.

**Fig. 3:**
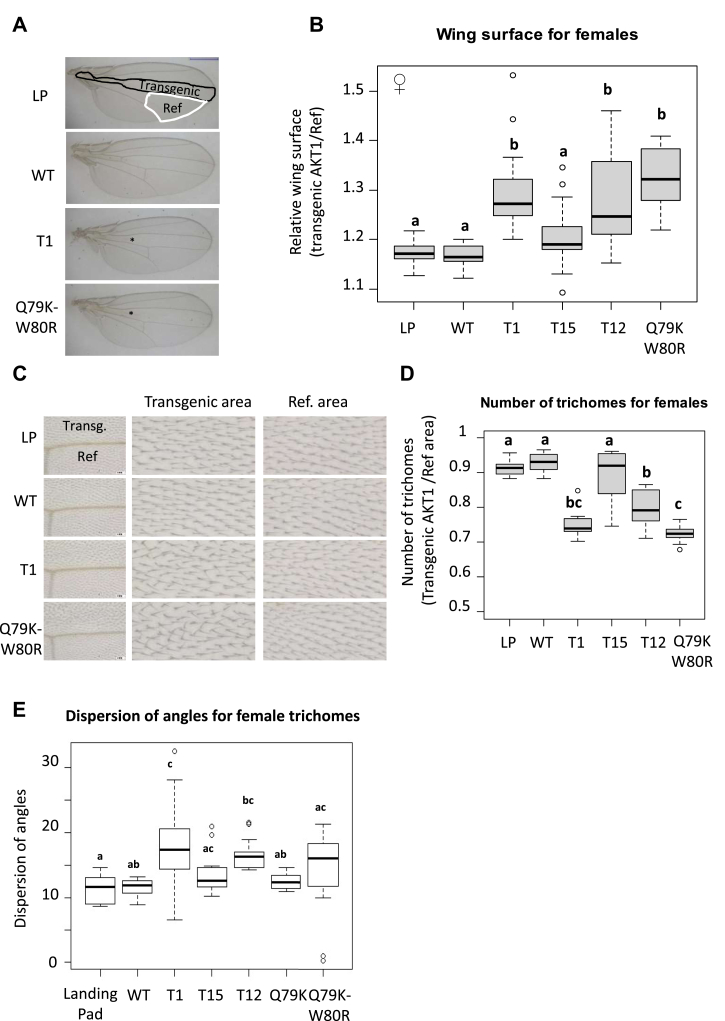
**Expression of WT or mutated AKT1 in wing imaginal disc affects cell growth**. *A*, adult wings from female flies not expressing AKT1 (LP), or expressing WT, T1, or Q79K-W80R. The *asterisks* (∗) show the disappearance of a characteristic vein. The scale bar represents 500 μM. *B*, boxplot of the variation of female relative wing surfaces when WT or mutated *AKT1* are expressed compared to control condition (LP)). Specifically, we calculated for each wing, the ratio of the area of a wing section expressing transgenic AKT1 (delimited by the *white line* in Fig. 3*A*, *uppermost panel*) over the surface of the section not expressing the transgene (in *black* in Fig. 3*A*). In the transgenic area, different forms of AKT1 are expressed under *patched*-GAL4 control. Landing pad/LP: *patched*-GAL4/landing pad (no AKT1 expression); WT: *patched*-GAL4/UAS-AKT1-WT; T1: *patched*-GAL4/UAS-AKT1-T1; T15: *patched*-GAL4/UAS-AKT1-T15; T12: *patched*-GAL4/UAS-AKT1-T12; Q79K-W80R: *patched-*GAL4/UAS-AKT1-Q79K-W80R. n = 20 per genotype except n = 9 for Q79KW80R. *C*, adult wings from female flies not expressing AKT1 (LP), or expressing WT, T1, or Q79K-W80R. For each wing, two rectangles of the same size were defined: one in the transgenic area and one in the area where the transgene was not expressed (Ref). *D*, boxplot of variation of female wing surfaces when WT or mutated *AKT1* are expressed compared to control condition (no AKT1 expression, LP). For each wing, the number of trichomes in the transgenic (AKT1 expression) section and in the section with no transgene expression (Ref) was counted and the ratios calculated. *E*, Dispersion of trichomes. The orientation of each trichome in the area expressing transgenic AKT1 was measured with ImageJ (directionality). *Box plots* represent the dispersion of the trichome angles for female for different genotypes. For each boxplot, the extreme points represent the 5th and 95th percentiles, whereas the *box* represents the 25th to 75th percentiles, with the median indicated in *black*. Letters *A*, *B*, and c refer to statistical categories in a Tukey test.

The wings in *Drosophila* are also an excellent tool for studying the impact of mutated forms of a protein on cell division and growth by estimating the density of trichomes on the wing surface. For this, we expressed the AKT1 variants in a section of the wings using the *patched*-GAL4 (*ptc-*GAL4) driver (Fig. 3*A*). Figure 3*B* clearly shows that, in females, the ratio of the surface of the section expressing human AKT1 (transgenic) over the reference surface (Ref) of the wing was similar to the condition LP (*i*.*e*.*,* no expression of exogenous AKT1). By contrast, this ratio increased in the fly lines where mutated AKT1 variants were expressed, reaching a maximum for the protein bearing the two pathogenic variants (*i*.*e*.*,* Q79K-W80R). To assess whether this increase of the surface was driven by cell proliferation in the wing primordia (more cells) or to the presence of larger cells (growth), we assessed cell number by evaluating the density of trichomes present in a given area of the wing section overexpressing AKT1 compared to a reference area (no AKT1 overexpression) covering the same surface. Since each cell contains only one trichome, trichome density directly reflects cell density, thereby indicating whether the cells are larger or smaller. Figure 3*C* shows that overexpression of mutated AKT1 leads to the presence of a smaller number of trichomes per surface unit than in the presence of WT1 AKT1 or in the LP (no overexpression) condition. This is particularly obvious in Figure 3*D*, which displays the number of trichomes present in a given surface in the wing section expressing transgenic AKT1 over to the number of trichomes in a reference area (no AKT1 overexpression). Specifically, trichome density was the highest in the LP and WT conditions and decreased when mutated AKT1 was expressed, reaching its smallest value for the double point-mutated (Q79K-W80R) protein. Similar results were obtained for males (Supplemental Fig. S3). Finally, we analyzed the dispersion of the trichomes. For this, we used ImageJ’s directionality plug-in that allowed us to infer the preferred orientation of the trichomes present in the input image. Specifically, we compared the dispersion of trichome angles for the different genotypes. The wings expressing the mutated AKT1 forms displayed higher dispersion values than those expressing WT AKT1 or the LP reference (Fig. 3*E*). These observations are in line with our results showing polarity alterations in ovarian follicle cells overexpressing mutated AKT1 (*i*.*e*.*,* abnormal morphology and nuclei much closer to the apical extremity than those expressing WT AKT1).

Altogether, these results indicate that human mutated forms of AKT are hyperactive in *Drosophila* and point to phenotypic effects relevant to cancer.

### Proteomic Analyses Point to Metabolic, Signaling, and Cytoskeletal Dysregulations

**Fig. 4:**
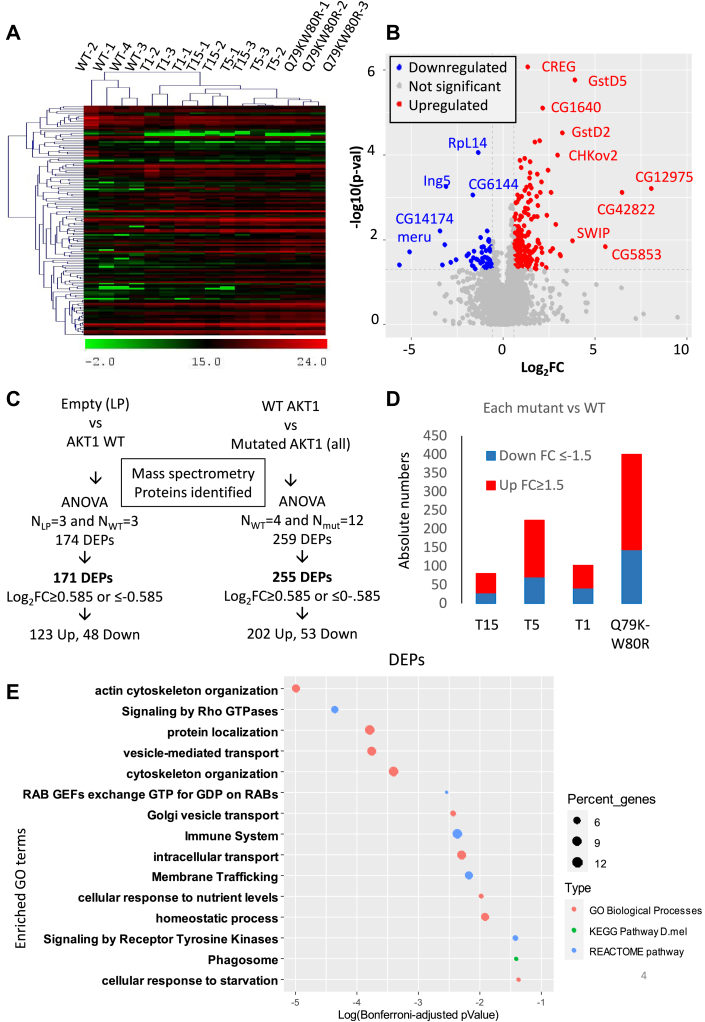
**Differential proteomic landscape of WT *versus* mutant AKT1 Drosophila ovarian follicle cells**. *A*, hierarchical clustering of DEPs in *Drosophila* expressing WT or mutated AKT1. *B*, volcano plot highlighting significantly upregulated (in *red*) and downregulated (in *blue*) proteins between WT or mutated AKT1. *C*, for each condition follicle cells of the progeny of three independent crosses underwent MS. The comparison “WT AKT1 *versus* LP”, yielded 174 were DE, of which 171 had a Log2FC of variation equal or greater than 0.585 in both directions (DEPs). For all mutants AKT1 (n = 12) *versus* WT AKT1 (n = 4), 259 proteins were DE, of which 255 had a Log2FC of variation equal or greater than 0.585 in both directions (DEPs). *D*, histogram represents upregulated (in *red*) and downregulated (in *blue*) proteins for each mutant *versus* WT AKT1. *E*, bubble plot of an ORA of the 255 DEPs in all mutants versus WT AKT1 described in Fig. 1*A*. DEP, differentially expressed protein; Log2FC, log2fold change; LP, landing pad; MS, mass spectrometry; ORA, overrepresentation analysis.

We used MS to explore the impact of AKT1 hyperactivation on the proteome of *Drosophila* follicle cells. For this, we used five lines (one overexpressing WT and four overexpressing hyperactive forms of AKT1) and compared them to the reference LP line (Fig. 4). Figure 4*A* displays a heat map showing that the biological replicates corresponding to the same conditions (namely, WT, T1, etc) clustered as expected. This is somewhat corroborated by a principal component analysis showing that the first principal component discriminates the WT from T1, T5, and Q79KW80R, while T15 appears scattered over the span covered by T1 and T5 (Supplemental Fig. S4, *A* and *B*). The distribution of log_2_-transformed protein intensities shows a symmetrical and centered profile around zero, indicating effective normalization and the absence of major systematic bias across the dataset (Supplemental Fig. S4*C*). Figure 4*B* represents a volcano plot highlighting significantly upregulated and downregulated proteins upon overexpression of WT and all of the mutated AKT1 versions considered together (allmut) to increase statistical power (*i*.*e*.*,* n = 4 *versus* n = 12). A similar analysis for the comparison WT *versus* LP (n = 3 *versus* n = 3 respectively, Fig. 4*C*) is available in the Figure S4*D*. These analyses uncovered 174 differentially expressed proteins (DEPs, *p* < 0.05) between *Drosophila* lines expressing WT AKT1 and LP and 259 DEPs (*p* < 0.05) between *Drosophila* expressing WT and all of the mutated AKT1 versions (allmut) (Fig. 4*C*). For subsequent analyses, we focused on the 171 proteins displaying a Log2FC ≥ 0.585 or ≤ −0.585, which corresponds to a FC ≥ 1.5 in either direction (DEPs) between *Drosophila* expressing WT AKT and LP and 255 DEPs between *Drosophila* expressing the WT and the mutated AKT1 versions. The former proteins (WT *versus* LP) included 48 upregulated and 123 downregulated proteins (Supplemental Table S1). The comparison (allmut *versus* wt) included 202 upregulated and 53 downregulated proteins (Supplemental Table S2, some examples of annotated spectra can be found in the Fig. S5).

When considering the DEPs for each mutant *versus* the WT (n = 3 *versus* n = 4 for each), we noted that their number vary from one mutated form to another, with T15 displaying the lowest number of DEPs whereas the double mutant had the strongest impact (Fig. 4*D*).

Next, we explored the cellular pathways responding to WT AKT1 overexpression (AKT1 *versus* LP) and in the mutants with respect to the WT condition (pooled analysis, *i*.*e*.*,* n = 4 *versus* n = 12) with PAthway, Network and Gene set Enrichment Analysis. This analysis pointed to several pathways significantly affected by WT AKT1 overexpression, such as glycolysis (consistent with our previous findings (32)) RNA degradation and lysosomal-related processes (corrected *p* values <0.05, Supplemental Table S3). DEPs between tissues expressing WT and mutated AKT1 were enriched in factors involved in Rho GTPase signaling, autophagy, cytoskeleton organization, and transport-related processes, among others (Fig. 4*E*).

### Transcriptomics Reveals Instances of Gain of Function but also Loss of Function

**Fig. 5:**
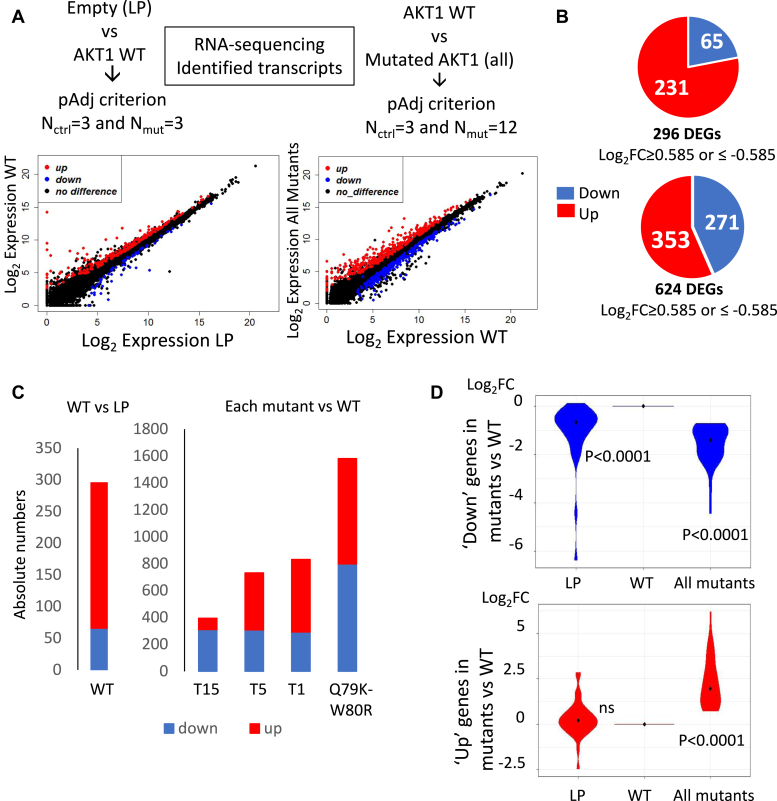
**Analysis of RNA-Seq data from follicle cells**. *A*, for each condition, follicle cells from the progeny of three independent crosses underwent RNA-Seq. The scatter plot shows the log2 gene expression values for the relevant comparisons. *Red and blue dots* stand for downregulated and upregulated genes between the different conditions, respectively. *B*, for each comparison a pie-chart represents the number of genes upregulated (in *red*) or downregulated (in *blue*). For the comparison LP *versus* WT AKT1, 296 genes had a Log2FC of variation equal or greater than 0.585 in both directions (DEGs) and were considered to be differentially expressed (231 up and 65 down). For WT AKT1 *versus* all mutants AKT1, 624 genes were differentially expressed (353 up and 271 down). *C*, histograms represent upregulated and downregulated genes for each comparison: WT AKT1 *versus* LP, T15 *versus* WT AKT1, T5 *versus* WT AKT1, T1 *versus* WT AKT1, and Q79KW80R *versus* WT AKT1. *D*, violin plots of the logFC levels for the 79 DEGs common to all the comparisons between *Drosophila* expressing WT and each mutated AKT1 version. DEG, differentially expressed gene; Log2FC, log2fold change; LP, landing pad.

To complete our exploration of the events dependent on AKT1 hyperactivation, we also performed an RNA-Seq analysis, using follicle cells from *Drosophila* lines overexpressing WT and the same four hyperactive forms of AKT1 (*versus* the reference LP line) (Fig. 5). RNA-Seq analysis uncovered 657 differentially expressed genes (DEGs, *p* < 0.05), between cells expressing WT AKT and LP (n = 3 *versus* n = 3, Figs. 5*A*) and 1082 DEGs between *Drosophila* expressing WT and mutated AKT1 versions (n = 3 *versus* n = 12, respectively). Next, we focused on the 296 genes displaying a Log2FC ≥ 0.585 or ≤ −0.585, which corresponds to a FC ≥ 1.5 in either direction (DEGs) between *Drosophila* expressing WT AKT and LP and 624 DEGs between *Drosophila* expressing WT and the mutated AKT1 versions (Fig. 5*B*, Supplemental Tables S4 and S5). In addition, we performed qRT-PCR experiments on nine selected DEGs between the WT and the T5 and Q79K-W80R conditions (*e*.*g*.*,* Pak3, Cib, Map205, Mhc,.) and found a strong correlation (R = 0.92) between the FC_RNA-Seq_ *versus* FC_RT-qPCR_ (Supplemental Fig. S6). The DEGs (WT *versus* LP) included 231 upregulated and 65 downregulated genes. The comparison (allmut *versus* wt) included 353 upregulated and 271 downregulated genes (Fig. 5*B*). When considering the number of DEGs for each mutant *versus* the WT (n = 3 *versus* n = 3 for each), we noted that the number of DEGs differed from one mutant to another: T15 displayed the lowest number of DEGs and the double mutant the highest, consistent with the proteomic results (Fig. 5*C*). Mutant T15 also displayed a different proportion of upregulated and downregulated genes, the latter being more represented.

A combined “metagene” analysis of the log2FC levels for the 79 DEGs common to all the comparisons between *Drosophila* expressing WT and each mutated AKT1 version showed that, on average, genes downregulated by the mutated forms compared to WT AKT1 were also downregulated in the LP condition (Supplemental Table S6). The comparison of the average value of the log2FC of the mutants *versus* WT or LP *versus* WT with respect to the reference value 0 (*i*.*e*.*,* no change, using a one-sample *t* test) produced *p* values <0.0001 in both cases. That is, such genes are upregulated by WT AKT1 and this effect disappears in the mutated conditions. This points to a potential loss of function of mutated AKT1 concerning the regulation of such specific genes. Regarding the genes upregulated by the mutated forms *versus* WT AKT1, the average expression of this gene subset was not affected by the expression of WT AKT1 as compared to LP, suggesting the existence of an expected gain of function of the mutated forms for this gene subset (Fig. 5*D*).

### Linking Proteomics and Transcriptomics Through Bioinformatics

Next, we identified the transcripts/genes corresponding to the 255 DEPs, in our RNA-Seq data. Interestingly, most of the genes were not significantly deregulated at the transcript level. This is consistent with our previous findings involving proteomic and transcriptomic comparisons of the effect of siRNAs targeting AKT1 *versus* a control ovary-derived murine cell line. Indeed, most DEProts had a transcript identified in the RNA-Seq data but only 10% of the latter were significantly deregulated (30).

**Fig. 6:**
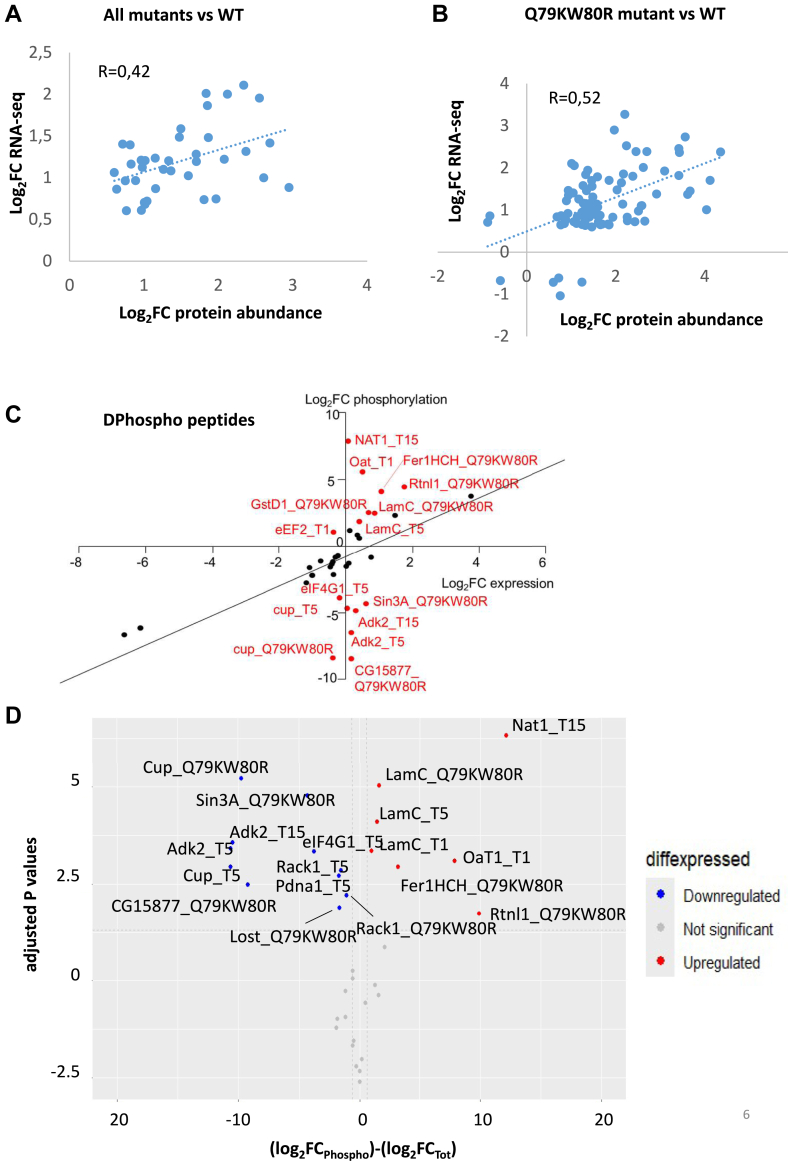
**Correlation between MS and RNA-Seq data and phosphopeptides analysis**. *A*, fold change correlation between mass spectrometry and RNA-Seq data for all mutants *versus* WT AKT1. *B*, fold2change correlation between mass spectrometry and RNA-Seq data for Q79K-W80R mutant *versus* WT AKT1. *C*, identification of the peptides differentially phosphorylated in mutants *versus* WT AKT1. The scatterplot of the Log2FC values resulting from DEPs and differentially phosphorylated peptides reveals two distinct populations of phosphorylated peptides. In *black*, the peptides displaying similar variations of phosphorylation and expression were not considered to be differentially phosphorylated. In *red*, the peptides displaying a variation of phosphorylation independent of the variation of the expression of the relevant proteins were considered as DPhospho peptides. *D*, volcano plot highlighting significantly up (*in red*) and down (*in blue*) phosphorylated in mutants *versus* WT AKT1. DEP, differentially expressed protein; MS, mass spectrometry; Log2FC, log2fold change.

In *Drosophila*, only 48 proteins matching DEGs had a Log2FC ≥ 0.585 or ≤ −0.585, out of which 47 proteins changed their expression in the same direction as their corresponding mRNAs (Supplemental Table S7). Again, this is consistent with our results in the mammalian system where less than 7% of proteins changed in the same direction as the corresponding transcripts (30). This points to a translational-level dysregulation (that could be mediated at least in part by the modulation of eEF2 and eIF4G1, see below). However, we cannot exclude technical limitations such as the existence of different detection thresholds and dynamic ranges in the proteomic and transcriptomic experiments. Figure 6*A* shows a positive and significant correlation (R = 0.42) between the FC observed in RNA-Seq and MS experiments for 38 proteins/RNAs after having removed 10 outliers (in the RNA-Seq or protein data) detected using the Iglewicz and Hoaglin's test (setting the modified Z score ≥3.5, http://contchart.com/outliers.aspx). We performed a similar analysis using data from Q79KW80R mutant (the strongest one, Fig. 6*B*). In this mutant, we found 108 DEPs that matched 108 DEGs (Supplemental Table S8). After removing 19 outliers, we found a stronger correlation (R = 0.52) between the FC observed in the RNA-Seq and MS experiments for the remaining 89 proteins/RNAs.

MS also uncovered peptides carrying posttranslational modifications. We used this information to identify potential differentially phosphorylated proteins (DPhospho-peptides) between the WT (n = 4) and each of the mutated conditions (n = 3) (although no phospho-peptide enrichment step was involved). MS data over all of the mutated conditions pointed to 36 phosphorylated peptides. To assess which of them were differentially phosphorylated, that is, not simply following the difference in protein expression, we computed the log2 of the ratios of the expression of the phospho-peptides in the mutated over the WT condition. We performed the same calculation for the expression levels of the corresponding proteins (30). We detected the DPhospho-peptides as the outliers using the Iglewicz and Hoaglin's test (setting the modified Z score ≥1, http://contchart.com/outliers.aspx). Fifteen peptides were found as “significant outliers” and thus considered as DPhospho-peptides (Fig. 6*C*). Another analysis involved a volcano plot highlighting significantly up (in red) and down (in blue) phosphorylated in mutants *versus* WT AKT1 is displayed in Figure 6*D* (Supplemental Table S9, some examples of annotated spectra can be found in Supplemental Fig. S7). In what follows, we focused on those peptides common to both analyses. They include peptides derived from cup, LamC and Adk2, which were detected in at least two mutated conditions (high confidence). Other interesting hits, appearing only in one condition and deserving further scrutiny, include eIF4G1, Oat, Rtnl1, Fer1HCH, Nat1, and Sin3A. Of note, the double mutated condition Q79K-W80R displayed the highest number of DPhospho-peptides. This correlates with its activity, which was the strongest among all of the mutated proteins studied. The differential phosphorylation of the peptides mentioned above (in addition to AKT1 itself), can be explained at least in part by the presence of 16 differentially expressed kinases and 1 DE phosphatase that we found in our MS data. We also explored the sequences of the relevant peptides using the Netphos prediction tool to infer potential upstream kinases. Several of the DPhospho-peptides could be potential targets of CKI/II, GSK3, and p38MAPK among others kinases (Supplemental Table S9).

**Fig. 7:**
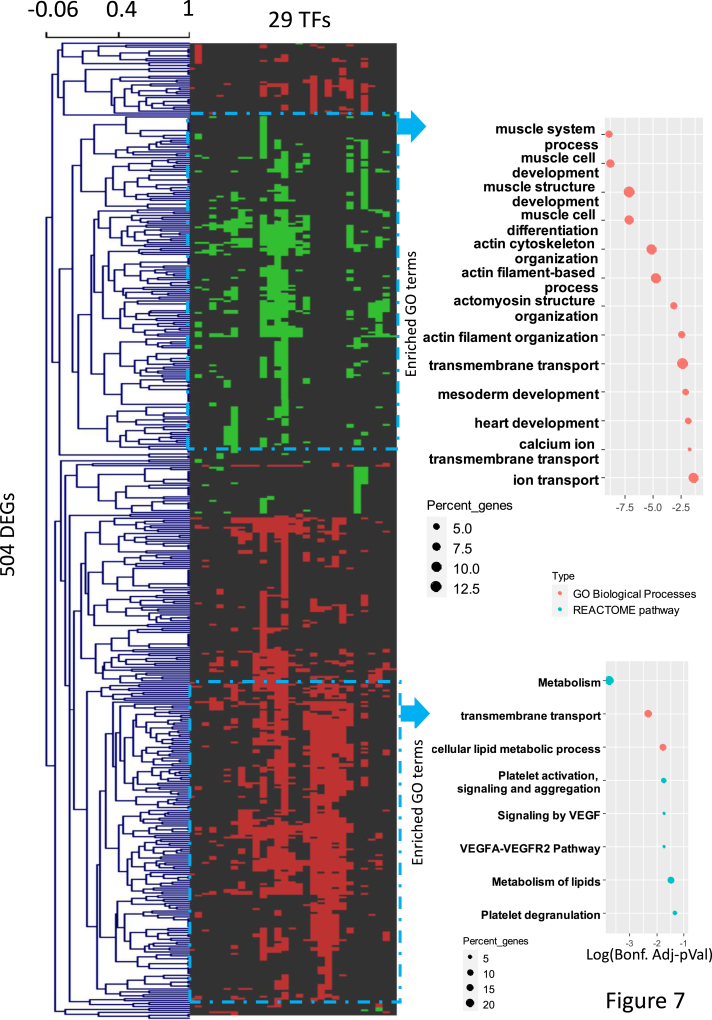
**Heat map of a two-way hierarchical clustering of 29 TFs identified as potential regulators of 504 DE genes**. Details of the selection process of both TFs and DE genes involved in the analysis are described the Experimental Procedures and Results sections. Two main groups of DE genes are highlighted and bubble plots of ORAs of the corresponding DEGs are shown on the *right*. The first one involved 174 genes downregulated by mutated AKT, and the second cluster involved 162 genes upregulated (Tables S11 and S12, respectively). ORA, overrepresentation analysis; TF, transcription factor.

The transcriptomic impact of AKT1 hyperactivation can be due to modulation of the expression of a set of TFs and/or to their posttranslational modification (even if they are not DE). Among the DE TFs, we found Brickwall (Brwl), Armadillo (arm), kayak (kay), Lipin (Lpin), and Misexpression suppressor of ras 3. In order to detect non-DE TFs (*i*.*e*.*,* at the protein level), the targets of which in other tissues are DE upon AKT1 hyperactivation, we explored the 624 DEGs using FlyEnrichR. Specifically, we conducted an ORA of the DEGs to uncover TFs the targets of which were statistically overrepresented. This analysis yielded a total of 40 TFs which might be direct or indirect targets of AKT1 or downstream of GSK3 (shaggy) in *Drosophila* follicle cells (DroID analysis, http://www.droidb.org/) (33). We focused on 29 TFs expressed in the ovary according to our MS or RNA-Seq data (Supplemental Table S10). Then, we generated a matrix containing one column for each of the 29 TFs, with 504 rows representing the relevant DE genes. The regulation or not of the i-th DEG by the j-th TF was encoded in a binary fashion (1 or 0, respectively (7)). Finally, a two-way hierarchical clustering was performed with MeV 4.8 (http://www.tm4.org/mev.html (34), using Pearson Correlation as a measure of similarity and average linkage) (Fig. 7). We found two main clusters, in terms the number of genes coregulated by a series of TFs. The first one contained a series of genes downregulated by mutated AKT, which are involved in actin cytoskeleton, ion, and other transport processes, etc (Supplemental Table S11). The second cluster contained upregulated genes involved in lipid metabolism and VEGF signaling (Supplemental Table S12).

**Fig. 8:**
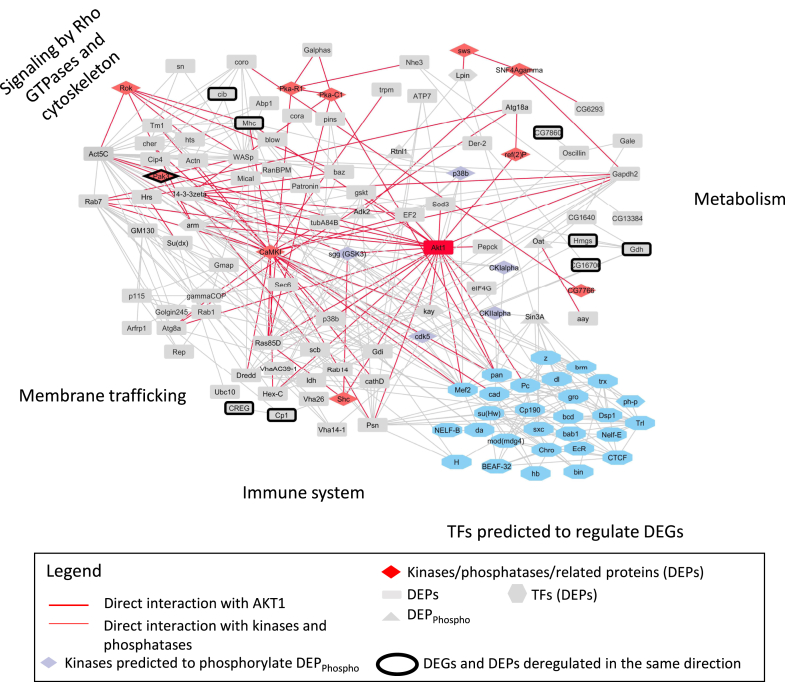
**Network of interactions linking mass spectrometry and RNA-Seq results**. The network was constructed using Cytoscape 3.8.0 (http://www.cytoscape.org) (27). The pathways shown to be significantly affected by WT AKT1 overexpression were derived from the PANGEA analysis of 255 DEPs (corrected *p* values < 0.05, Table S3). Genes/proteins deregulated at both transcriptomic and proteomic levels have been highlighted by a *thick black line*. DEP, differentially expressed protein; PANGEA, PAthway, Network and Gene set Enrichment Analysis.

Finally, we built a network of protein–protein interactions (PPIs) involving DEPs including the proteins containing the DPhospho-peptides, the four kinases predicted to phosphorylate the DPhospho-peptides and the DE kinases+phosphatases (Fig. 8). This figure shows an extremely rich network of PPIs and the hub status of AKT1. The 29 TFs described above that regulate 504 DEGs are also highly connected (Fig. 8, see discussion).

## Discussion

In this study, we have used a series of *Drosophila* lines overexpressing WT and five hyperactive forms of AKT1 in follicle cells (and a reference LP line), to get insights into the cellular and molecular events behind JGCT formation. As mentioned in the Results section, the number of DEGs differed from one mutated form to another, with T15 (*i*.*e*.*,* the smallest insertion) displaying the lowest number and the one carrying the two-point variants Q79K-W80R, the highest, which correlates with their phenotypic effects.

Interestingly, we found at the transcriptional level that a series of genes indirectly upregulated by WT AKT1 were less well activated by the mutated forms. This downward U-shape suggests the existence of a potential loss of function of mutated AKT1 impacting the transcriptional regulation of such genes. This phenomenon is well known to pharmacologists and can appear in cell signaling and transcription (35). For another gene subset, we could gather evidence for a clear gain of function of the mutated forms (Fig. 5*D*), consistent with the expected pathogenic mechanism for oncogenic proteins ((36) and references therein). These results point to an unanticipated mechanistic complexity because the classical assumption for “activating” oncogenic pathogenic variants is that of a gain of function, as has been proposed for the classical E17K AKT1 variant (37).

The MS experiments that we performed were not designed to focus on DPhospho-peptides because they did not involve any phospho-peptide enrichment step. However, we were able to detect a series of DPhospho-peptides. Our analysis yielded a few candidate hyperphosphorylated proteins such as LamC (nuclear lamin), eIF4G1 (translation initiation), among others. Other peptides such as those derived from cup (mRNA localization in the oocyte) or Adk2 (purine metabolism) showed a strongly decreased phosphorylation suggesting that they might be potential targets of phosphatases that could be in turn AKT1 direct/indirect targets. Sin3A, a member of the Sin3 family of proteins linked to tumorigenesis was also found in this category. They are thought to regulate gene expression through their role as histone deacetylases and Sin3a is an important mediator of the effects of the oncogenic Ret receptor in a fly model of multiple endocrine neoplasia type 2. The link between AKT1 and Sin3A requires specific attention because the latter is known to modulate tumorigenesis by regulating a module of genes involved in cell invasion (38). On general grounds, our results confirm the implication of AKT1 in the regulation of translation (39) and points to a potential involvement of the kinase in the global regulation of gene expression through direct or indirect phosphorylation of LamC (40).

The comparison RNA-Seq *versus* MS shows that most of the transcripts/genes corresponding to the 255 DEPs were not DE. Indeed, only 20% of DEPs displayed a consistent differential expression at the mRNA level. As we have previously shown in murine granulosa cells, we invoke a layer posttranscriptional events affecting translation or stability of such proteins to explain the existence of DEPs in the absence of DE at the transcriptional level (30)). This is in line with the fact the AKT1 modifies the phosphorylation status of eIF4G1.

Figure 8 displays an interaction map of several differentially regulated players (proteins, genes, phospho-peptides) upon the action of mutated AKT1 forms and illustrates the diversity of processes and pathways that are perturbed. Interestingly, we found that calcium/calmodulin-dependent protein kinase 1 displays, along with AKT1, the maximum node degree in the PPI network. This kinase is upregulated by about 2-fold in the mutant follicle cells. A potential direct or indirect interaction of both kinases in the context of *Drosophila* cells (and potentially in the context of JGCTs) deserves further studies. In line with this, we found in a previous study using mammalian cells that CALM1 could represent a potential direct target of AKT1. Indeed, its phosphorylation level was shown too sensitive to AKT1 (depleted by knock down) but further work is required to explore the biological relevance of this findings (30). To explore how mutated AKT1 orchestrates the transcriptional changes we observed, we focused on TFs affected by AKT1 overexpression at the protein level. However, posttranslational modifications of a series of TFs (missed in our MS data) without differential expression can also be at work. To take this possibility into account, we performed *in silico* analyses to identify TFs the targets of which are DE according to our RNA-Seq data. Figure 8 also shows that such TFs are densely connected among them and with the DE TFs. They are also connected with AKT1 and calcium/calmodulin-dependent protein kinase 1. The high degree of connectivity observed points to causal relationships among the relevant genes and proteins and also point to a link with a perturbation of cell polarity by the AKT1 pathogenic variants. This requires further investigation. As shown in the figure, DEGs potentially regulated by non-DE TFs basically cluster into two main groups involved in cytoskeleton-based transactions and different facets of metabolism, in agreement with previous knowledge on AKT1 ((30) and references therein). This lends credence to the implication of such TFs on the regulation of the relevant DEGs.

Interestingly, we observed an upregulation of multiple glutathione S-transferase (GST) genes, including GstD2, GstD5, GstD9, and GstE1, suggesting a robust cellular response to oxidative stress that might result from AKT1 hyperactivation. Indeed, enhanced AKT1 signaling is known to increase anabolic processes and mitochondrial activity, leading to elevated reactive oxygen species levels. To counteract the potential damage from reactive oxygen species, cells upregulate detoxifying enzymes such as GSTs. Notably, GstD2 has been identified as part of a general stress response in *Drosophila*, being upregulated under various stress conditions including oxidative stress and aging. Similarly, GstE1 is involved in detoxification processes that protect against oxidative damage. The induction of these GSTs underscores the cell's adaptive mechanisms to maintain redox homeostasis in the face of metabolic stress induced by AKT1 hyperactivation (41, 42).

Regarding the phenotypic effects of the pathogenic variants on the wings of *Drosophila*, we found a decreased density of trichomes when mutated AKT1 was expressed with respect to the LP and WT conditions. This suggests that the increase of the wing surface is more likely due to an increase of cell size (growth) than to cell proliferation. Indeed, AKT indirectly activates mTOR kinase which promotes cell growth. Specifically, AKT inhibits the TSC1-TSC2 complex entailing stimulation of mTOR, which promotes cell growth (size) and proliferation (number) via the regulation of ribosomal biogenesis and mRNA translation (mTOR phosphorylates p70S6K and several eIF4E binding proteins (43)).

As shown in the results section, trichome density reached its smallest value for the mutant Q79K-W80 R confirming that it is the most hyperactive variant tested. Upregulation of the cytoskeletal components Cher (Filamin) and Act5C (actin 5C) aligns with the observed trichome misorientation. This causes changes in actin cytoskeleton dynamics that can lead to impaired planar cell polarity, explaining the increased trichome angle dispersion (44, 45, 46).

The expression of the various mutated AKT1 forms in ovarian follicle cells led to abnormal cell morphology correlating with the degree of activation (as shown in previous experiments). The nuclei appeared much closer to the basal cell pole than those expressing WT AKT1, which points again to a perturbation of cell polarity (Fig. 2*C*). Such cells can be considered as “epithelial cells” which are polarized along their apical–basal axis. We find in our MS data that Pak3 is activated (2X in cells expressing the mutated AKT versions). This is supposed to alter the specification of the apical domain identity in such cells. Indeed, it is known that Pak kinases can phosphorylate polarity proteins and their overactivation disrupts epithelial organization. This is consistent with the polarity abnormalities we observed in ovarian follicular cells (47). This is accompanied by a loss of apical polarization markers such as Crumbs, Par3/Bazooka, or ZO-1 (48). Consistently, we show a decreased expression of Baz in our MS data.

Our results also show that AKT is involved in conveying cell-fate information driving cell shape changes and movements. This is particularly so in the generation of the dorsal appendages (31). Indeed, we observed the presence of shorter and/or abnormal respiratory appendages in the embryos expressing mutated AKT1 (Fig. S2). Consistently, several transcripts and proteins involved in the morphogenesis of the dorsal appendages were deregulated by the mutants (31). For instance, cheerio/filamin (mentioned above), peanut, singed/fascin, kayak/fos, and fasciclin were found upregulated in our MS data.

Our data, taken altogether, map the *in vivo* molecular effects of AKT1 activation at the proteomic and transcriptomic levels in *Drosophila* follicle cells. The expression of mutated forms provokes the appearance of mutant phenotypes (ovary, wings) confirming their probable causality in pathology. We confirm and extend the role of this kinase in critical cancer-related cellular processes beyond cell growth and proliferation. Our experimental and *in silico* study provides a series of dysregulated pathways that deserve further investigation to shed light upon the implication of this kinase in oncogenic processes.

## Data Availability

RNA-Seq data were deposited in Gene Expression Omnibus (RRID: SCR_005012) (GSE251846). MS data are available via ProteomeXchange with identifier PXD060549.

## Supplemental Data

This article contains supplemental data.

## Conflict of Interests

The authors declare no competing interests.

## References

[1] Pectasides D., Pectasides E., Psyrri A. (2008). Granulosa cell tumor of the ovary. Cancer Treat. Rev..

[2] Young R.H., Scully R.E. (1992). Endocrine tumors of the ovary. Curr. Top. Pathol. Ergeb. Pathol..

[3] Fleming N.A., de Nanassy J., Lawrence S., Black A.Y. (2010). Juvenile granulosa and theca cell tumor of the ovary as a rare cause of precocious puberty: case report and review of literature. J. Pediatr. Adolesc. Gynecol..

[4] Kalfa N., Philibert P., Patte C., Ecochard A., Duvillard P., Baldet P. (2007). Extinction of FOXL2 expression in aggressive ovarian granulosa cell tumors in children. Fertil. Steril..

[5] Kalfa N., Ecochard A., Patte C., Duvillard P., Audran F., Pienkowski C. (2006). Activating mutations of the stimulatory g protein in juvenile ovarian granulosa cell tumors: a new prognostic factor?. J. Clin. Endocrinol. Metab..

[6] Bessière L., Todeschini A.L., Auguste A., Sarnacki S., Flatters D., Legois B. (2015). A hot-spot of in-frame duplications activates the oncoprotein AKT1 in juvenile granulosa cell tumors. EBioMedicine.

[7] Auguste A., Bessière L., Todeschini A.L., Caburet S., Sarnacki S., Prat J. (2015). Molecular analyses of juvenile granulosa cell tumors bearing AKT1 mutations provide insights into tumor biology and therapeutic leads. Hum. Mol. Genet..

[8] Gibson T.J., Hyvönen M., Musacchio A., Saraste M., Birney E. (1994). PH domain: the first anniversary. Trends Biochem. Sci..

[9] Warfel N.A., Niederst M., Newton A.C. (2011). Disruption of the interface between the pleckstrin homology (PH) and kinase domains of Akt protein is sufficient for hydrophobic motif site phosphorylation in the absence of mTORC2. J. Biol. Chem..

[10] Mahajan K., Mahajan N.P. (2012). PI3K-independent AKT activation in cancers: a treasure trove for novel therapeutics. J. Cell. Physiol..

[11] Yi K.H., Axtmayer J., Gustin J.P., Rajpurohit A., Lauring J. (2013). Functional analysis of non-hotspot AKT1 mutants found in human breast cancers identifies novel driver mutations: implications for personalized medicine. Oncotarget.

[12] Calleja V., Laguerre M., Parker P.J., Larijani B. (2009). Role of a novel PH-kinase domain interface in PKB/Akt regulation: structural mechanism for allosteric inhibition. Plos Biol..

[13] Pereira B., Chin S.F., Rueda O.M., Vollan H.K.M., Provenzano E., Bardwell H.A. (2016). The somatic mutation profiles of 2,433 breast cancers refines their genomic and transcriptomic landscapes. Nat. Commun..

[14] Lefebvre C., Bachelot T., Filleron T., Pedrero M., Campone M., Soria J.C. (2016). Mutational profile of metastatic breast cancers: a retrospective analysis. PLoS Med..

[15] Zehir A., Benayed R., Shah R.H., Syed A., Middha S., Kim H.R. (2017). Mutational landscape of metastatic cancer revealed from prospective clinical sequencing of 10,000 patients. Nat. Med..

[16] Chang M.T., Bhattarai T.S., Schram A.M., Bielski C.M., Donoghue M.T.A., Jonsson P. (2018). Accelerating discovery of functional mutant alleles in cancer. Cancer Discov..

[17] Yeh Y.-C., Ho H.L., Wu Y.C., Pan C.C., Wang Y.C., Chou T.Y. (2020). AKT1 internal tandem duplications and point mutations are the genetic hallmarks of sclerosing pneumocytoma. Mod. Pathol..

[18] Brunet A., Bonni A., Zigmond M.J., Lin M.Z., Juo P., Hu L.S. (1999). Akt promotes cell survival by phosphorylating and inhibiting a Forkhead transcription factor. Cell.

[19] Aoki M., Fujishita T. (2017). Oncogenic roles of the PI3K/AKT/mTOR axis. Curr. Top. Microbiol. Immunol..

[20] Mirzoyan Z., Sollazzo M., Allocca M., Valenza A.M., Grifoni D., Bellosta P. (2019). Drosophila melanogaster: a model organism to study cancer. Front. Genet..

[21] Bischof J., Maeda R.K., Hediger M., Karch F., Basler K. (2007). An optimized transgenesis system for Drosophila using germ-line-specific ϕC31 integrases. Proc. Natl. Acad. Sci. U. S. A..

[22] Gervais L., Claret S., Januschke J., Roth S., Guichet A. (2008). PIP5K-dependent production of PIP2 sustains microtubule organization to establish polarized transport in the Drosophila oocyte. Dev. Camb. Engl..

[23] Bryant Z., Subrahmanyan L., Tworoger M., LaTray L., Liu C.R., Li M.J. (1999). Characterization of differentially expressed genes in purified Drosophila follicle cells: toward a general strategy for cell type-specific developmental analysis. Proc. Natl. Acad. Sci. U. S. A..

[24] Schneider C.A., Rasband W.S., Eliceiri K.W. (2012). NIH Image to ImageJ: 25 years of image analysis. Nat. Methods..

[25] Dobens L.L., Shipman A., Axelrod J.D. (2018). FijiWingsPolarity: an open source toolkit for semi-automated detection of cell polarity. Fly (Austin).

[26] Szklarczyk D., Gable A.L., Lyon D., Junge A., Wyder S., Huerta-Cepas J. (2019). STRING v11: protein-protein association networks with increased coverage, supporting functional discovery in genome-wide experimental datasets. Nucleic Acids Res..

[27] Shannon P., Markiel A., Ozier O., Baliga N.S., Wang J.T., Ramage D. (2003). Cytoscape: a software environment for integrated models of biomolecular interaction networks. Genome Res..

[28] Taus T., Köcher T., Pichler P., Paschke C., Schmidt A., Henrich C., Mechtler K. (2011). Universal and confident phosphorylation site localization using phosphoRS. J. Proteome Res..

[29] Perez-Riverol Y., Bandla C., Kundu D.J., Kamatchinathan S., Bai J., Hewapathirana S. (2025). The PRIDE database at 20 years: 2025 update. Nucleic Acids Res..

[30] Elzaiat M., Herman L., Legois B., Léger T., Todeschini A.L., Veitia R.A. (2019). High-throughput exploration of the network dependent on AKT1 in mouse ovarian granulosa cells. Mol. Cell. Proteomics..

[31] Berg C.A. (2005). The Drosophila shell game: patterning genes and morphological change. Trends Genet..

[32] Hung Y.P., Teragawa C., Kosaisawe N., Gillies T.E., Pargett M., Minguet M. (2017). Akt regulation of glycolysis mediates bioenergetic stability in epithelial cells. eLife.

[33] Murali T., Pacifico S., Yu J., Guest S., Roberts G.G., Finley R.L. (2011). DroID 2011: a comprehensive, integrated resource for protein, transcription factor, RNA and gene interactions for Drosophila. Nucleic Acids Res..

[34] Saeed A.I., Sharov V., White J., Li J., Liang W., Bhagabati N. (2003). TM4: a free, open-source system for microarray data management and analysis. BioTechniques.

[35] Gibson T.J., Seiler M., Veitia R.A. (2013). The transience of transient overexpression. Nat. Methods.

[36] Pilsworth J.A., Todeschini A.L., Neilson S.J., Cochrane D.R., Lai D., Anttonen M. (2021). FOXL2 in adult-type granulosa cell tumour of the ovary: oncogene or tumour suppressor gene?. J. Pathol..

[37] Chen Y., Huang L., Dong Y., Tao C., Zhang R., Shao H., Shen H. (2020). Effect of AKT1 (p. E17K) hotspot mutation on malignant tumorigenesis and prognosis. Front. Cell. Dev. Biol..

[38] Das T.K., Sangodkar J., Negre N., Narla G., Cagan R.L. (2013). Sin3a acts through a multi-gene module to regulate invasion in Drosophila and human tumors. Oncogene.

[39] Ruggero D., Sonenberg N. (2005). The Akt of translational control. Oncogene.

[40] Gurudatta B.V., Shashidhara L.S., Parnaik V.K. (2010). Lamin C and chromatin organization in Drosophila. J. Genet..

[41] Gonzalez D., Fraichard S., Grassein P., Delarue P., Senet P., Nicolaï A. (2018). Characterization of a Drosophila glutathione transferase involved in isothiocyanate detoxification. Insect. Biochem. Mol. Biol..

[42] Enayati A.A., Ranson H., Hemingway J. (2005). Insect glutathione transferases and insecticide resistance. Insect. Mol. Biol..

[43] Cheung M., Testa J.R. (2013). Diverse mechanisms of AKT pathway activation in human malignancy. Curr. Cancer. Drug. Targets..

[44] Koehler S., Huber T.B., Denholm B. (2022). A protective role for Drosophila Filamin in nephrocytes via Yorkie mediated hypertrophy. Life Sci. Alliance..

[45] Sokol N.S., Cooley L. (1999). Drosophila filamin encoded by the cheerio locus is a component of ovarian ring canals. Curr. Biol..

[46] Ugrankar-Banerjee R., Tran S., Bowerman J., Kovalenko A., Paul B., Henne W.M. (2023). The fat body cortical actin network regulates Drosophila inter-organ nutrient trafficking, signaling, and adipose cell size. eLife.

[47] Ozdowski E.F., Gayle S., Bao H., Zhang B., Sherwood N.T. (2011). Loss of Drosophila melanogaster p21-activated kinase 3 suppresses defects in synapse structure and function caused by spastin mutations. Genetics.

[48] M A.-A., Elbediwy A., Foglizzo V., Fletcher G.C., Li V.S.W., Thompson B.J. (2018). Pak1 kinase maintains apical membrane identity in Epithelia. Cell Rep.

