# Supplementary material for: Deciphering the Impact of AKT1 Pathogenic Variants in Juvenile Granulosa Cell Tumors Using a Drosophila Model
Source: Mol Cell Proteomics. 2025 Nov 13;24(12):101466. doi: 10.1016/j.mcpro.2025.101466 (PMC12757477; doi:10.1016/j.mcpro.2025.101466)
Supplement: Table S12 [file mmc13.pdf]

Figure S1: Immunostaining of an egg chamber expressing WT or T5 mutant AKT1 under the control of the driver *traffic jam*-GAL4. Actin was stained with phalloidin (in red), the plasma membrane by Wheat Germ Agglutin (in red), AKT1 by immunostaining (in green, anti-phospho-S473 AKT1) and nuclei are stained with Hoechst. The white rectangles indicate the positions that are zoomed in on the panels below each relevant panel to show the localization of WT or mutated AKT1 and the mislocalization of the nuclei in the mutants. Scale bars indicate 20 µm.

Figure S2: Chorionic phenotypes resulting from mutated AKT1 expression. a. Mature eggs laid by females expressing WT or mutated AKT1 under the control of the driver *traffic jam*-GAL4. Different phenotypes were scored: shorter and/or fused dorsal appendages. Scale bars indicate 200 µm. **b-c.** Percentage of eggs in each category (normal or abnormal) laid by females expressing WT or mutant AKT1 at 22°C (**b**) or at 25°C with stronger expression of transgenic forms of AKT1 (**c**)**.**

Figure S3: Expression of WT or mutated AKT1 in wing imaginal disc affects cell growth. a. Adult wings from male flies not expressing AKT1 (LP), or expressing WT, T1 or Q79K-W80R. The asterisks (*) show the disappearance of a characteristic vein. Scale bar=500µM **b.** Boxplot of the variation of male relative wing surfaces when WT or mutated *AKT1* are expressed compared to control condition (no AKT1 expression, Landing pad (LP)). Specifically, we calculated for each wing, the ratio of the area of a wing section expressing transgenic AKT1 (delimited by the white line in Fig S3a, uppermost panel) over the surface of the section not expressing the transgene (in black in Fig S3a). In the transgenic area, different forms of AKT1 are expressed under *patched*-GAL4 control. Landing Pad: *patched*-GAL4/Landing pad (no AKT1 expression); WT: *patched*-GAL4/UAS-AKT1-WT; T1: *patched*-GAL4/UAS-AKT1-T1 ; T15: *patched*-GAL4/UAS-AKT1-T15 ; T12: *patched*-GAL4/UAS-AKT1-T12 ; Q79K-W80R: *patched*-GAL4/UAS-AKT1-Q79K-W80R. **c. Adult wings from male flies not expressing AKT1 (LP), or expressing WT, T1 or Q79K-W80R.** For each wing, two rectangles of the same size were defined: one in the transgenic area and one in the area where the transgene was not expressed (Ref). **d. Boxplot of variation of male wing surfaces when WT or mutated *AKT1* are expressed compared to control condition (no AKT1 expression, LP).** For each wing, the number of trichomes in the transgenic (AKT1 expression) section and in the section with no transgene expression (Ref) was counted and the ratios calculated.

Figure S4: **a-b Principal Component Analysis of DEPs in Drosophila expressing WT or mutated AKT1 with the three biological replicates of each condition averaged (a) or not (b).** The different conditions are represented as follows: WT in

black, T1 (=MUT-1) in red, T15 (=MUT2) in blue, T5 (=MUT3) in green and Q79KW80R (=MUT4) in pink. **c.** Histogram showing the distribution of log_2-transformed protein intensities for mutant versus WT conditions. The majority of proteins exhibit intensities centered around zero, indicating normalization of the data and comparable expression levels across samples. **d**. Volcano plot highlighting significantly up- (in red) and down (in blue) -regulated proteins between WT or LandingPad conditions.

Figure S5: Annotated MS/MS spectra for some protein identified on one peptide. Representative tandem mass spectrometry spectra showing peptide sequence coverage and fragment ion assignment (b- and y-ions) for 14-3-3-zeta (24652326), Actn (442614864), Hnf4 (241669014), Hex-C (10765475), Kay (221460401). For each example, sequence and -10LogP (localization confidence scores) are indicated above spectrum.

Figure S6: Correlation between RT-qPCR and RNAseq data. FoldChange correlation between RT-qPCR and RNAseq data for T5 and Q79KW80R.

Figure S7: Annotated MS/MS spectra of phosphopeptides. Representative tandem mass spectrometry spectra showing peptide sequence coverage and fragment ion assignment (b- and y-ions) for phosphopeptides identified from CUP (2213915), eEF2 (24585709), eIF4G1 (161076327), and LamC (531500). A few examples are presented, involving serine as the phosphorylation site. The position concerned is indicated by a blue arrow. -10LogP : localization confidence scores.

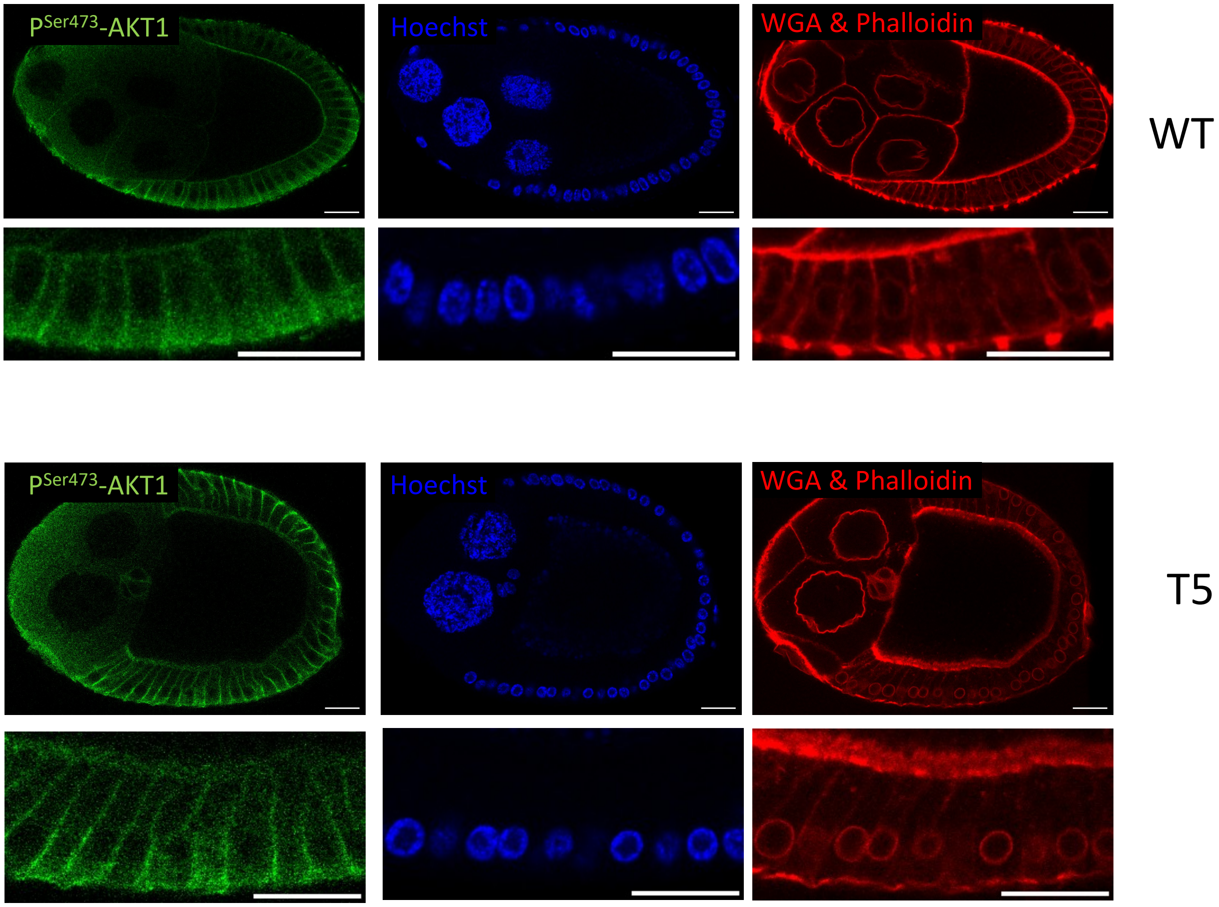

PSer473-AKT1
Hoechst
WGA & Phalloidin
WT
PSer473-AKT1
Hoechst
WGA & Phalloidin
T5

Figure S1

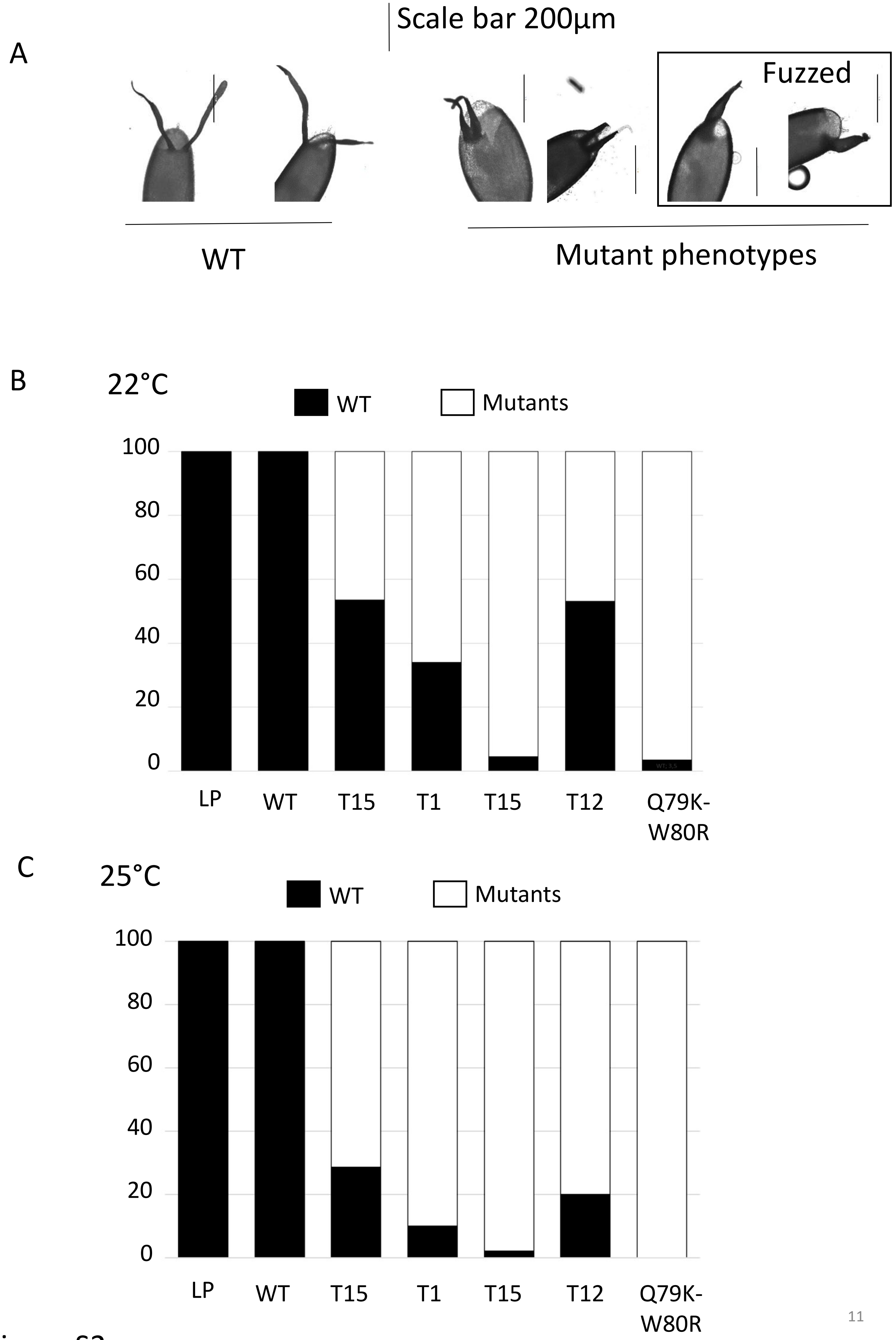
A
Scale bar 200µm
Fuzzed
WT
Mutant phenotypes
B
22°C
WT
Mutants
100
80
60
40
20
0
LP
WT
T15
T1
T15
T12
Q79K-W80R
C
25°C
WT
Mutants
100
80
60
40
20
0
LP
WT
T15
T1
T15
T12
Q79K-W80R

Figure S2

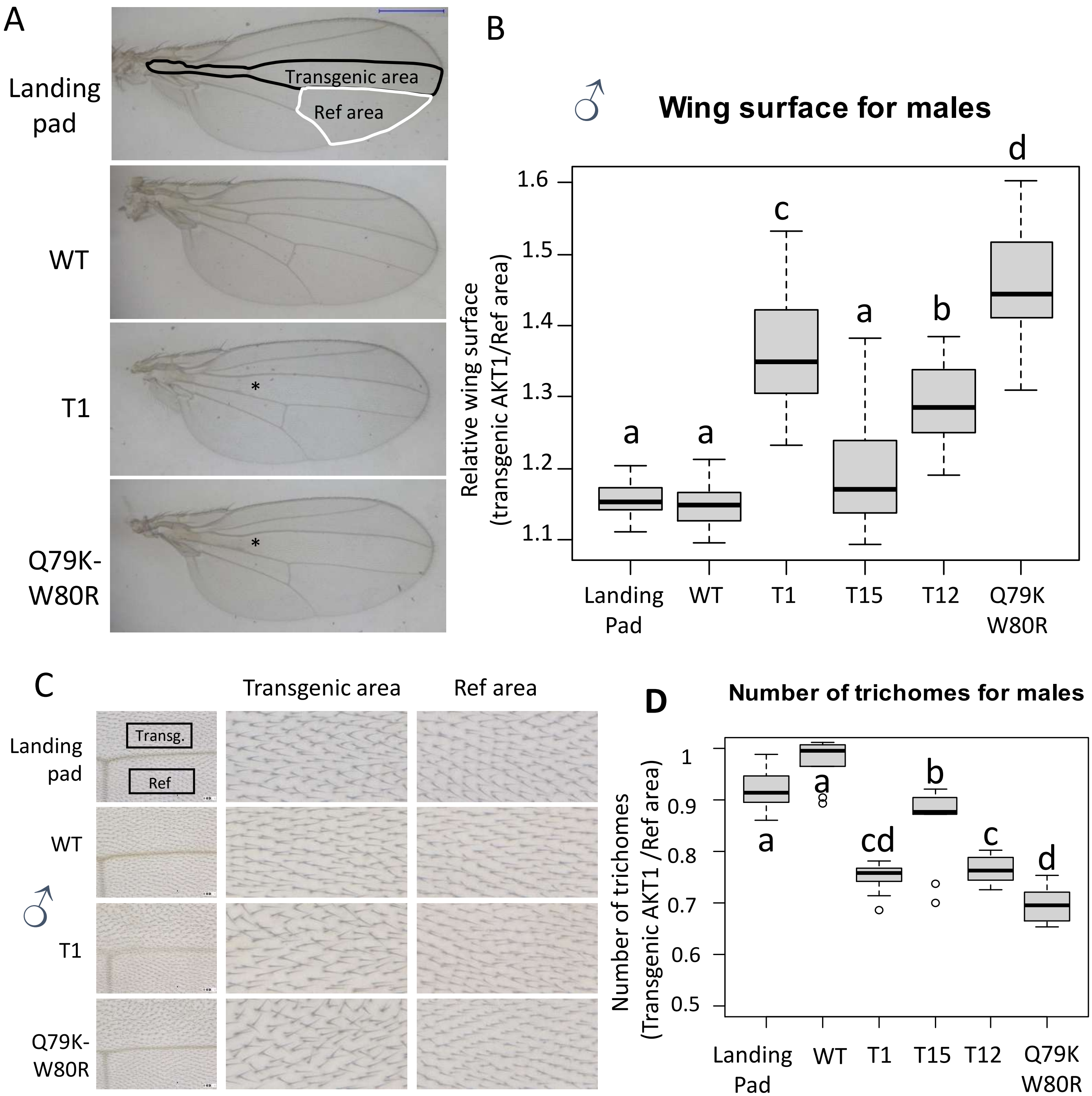
A
Landing pad
Transgenic area
Ref area
WT
T1
Q79K-W80R
B
Wing surface for males
Relative wing surface (transgenic AKT1/Ref area)
1.6
1.5
1.4
1.3
1.2
1.1
a
a
c
a
b
d
Landing Pad
WT
T1
T15
T12
Q79K W80R
C
Transgenic area
Ref area
Landing pad
Transg.
Ref
WT
T1
Q79K-W80R
D
Number of trichomes for males
Number of trichomes (Transgenic AKT1 /Ref area)
1
0.9
0.8
0.7
0.6
0.5
a
a
cd
b
c
d
Landing Pad
WT
T1
T15
T12
Q79K W80R

Figure S3

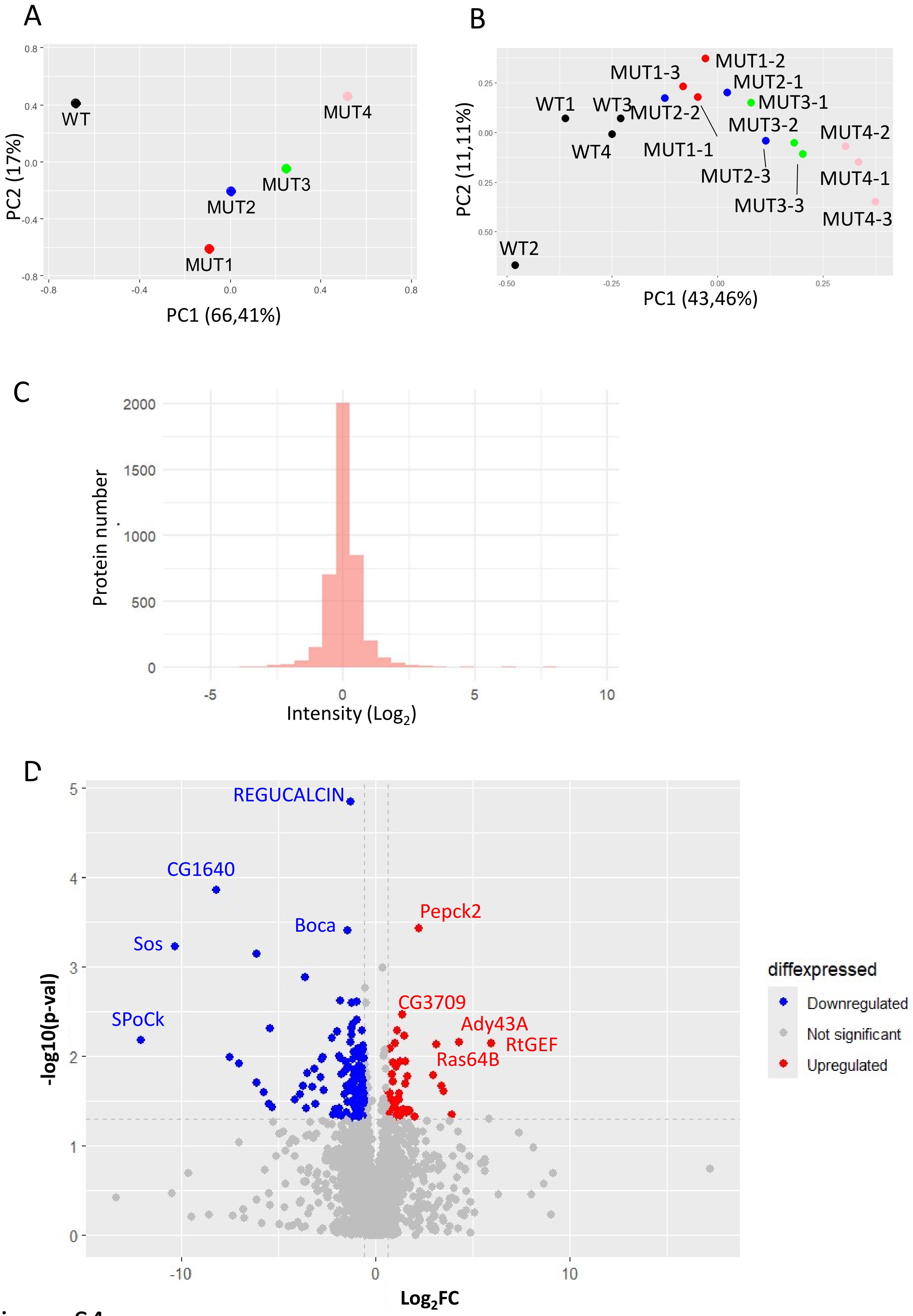

A
B
C
D
PC1 (66,41%)
PC2 (17%)
WT
MUT1
MUT2
MUT3
MUT4
PC1 (43,46%)
PC2 (11,11%)
WT1
WT2
WT3
WT4
MUT1-1
MUT1-2
MUT1-3
MUT2-1
MUT2-2
MUT2-3
MUT3-1
MUT3-2
MUT3-3
MUT4-1
MUT4-2
MUT4-3
Protein number
Intensity (Log2)
-log10(p-val)
Log2FC
REGUCALCIN
CG1640
Boca
Sos
SPoCk
Pepck2
CG3709
Ady43A
RtGEF
Ras64B
diffexpressed
Downregulated
Not significant
Upregulated

Figure S4

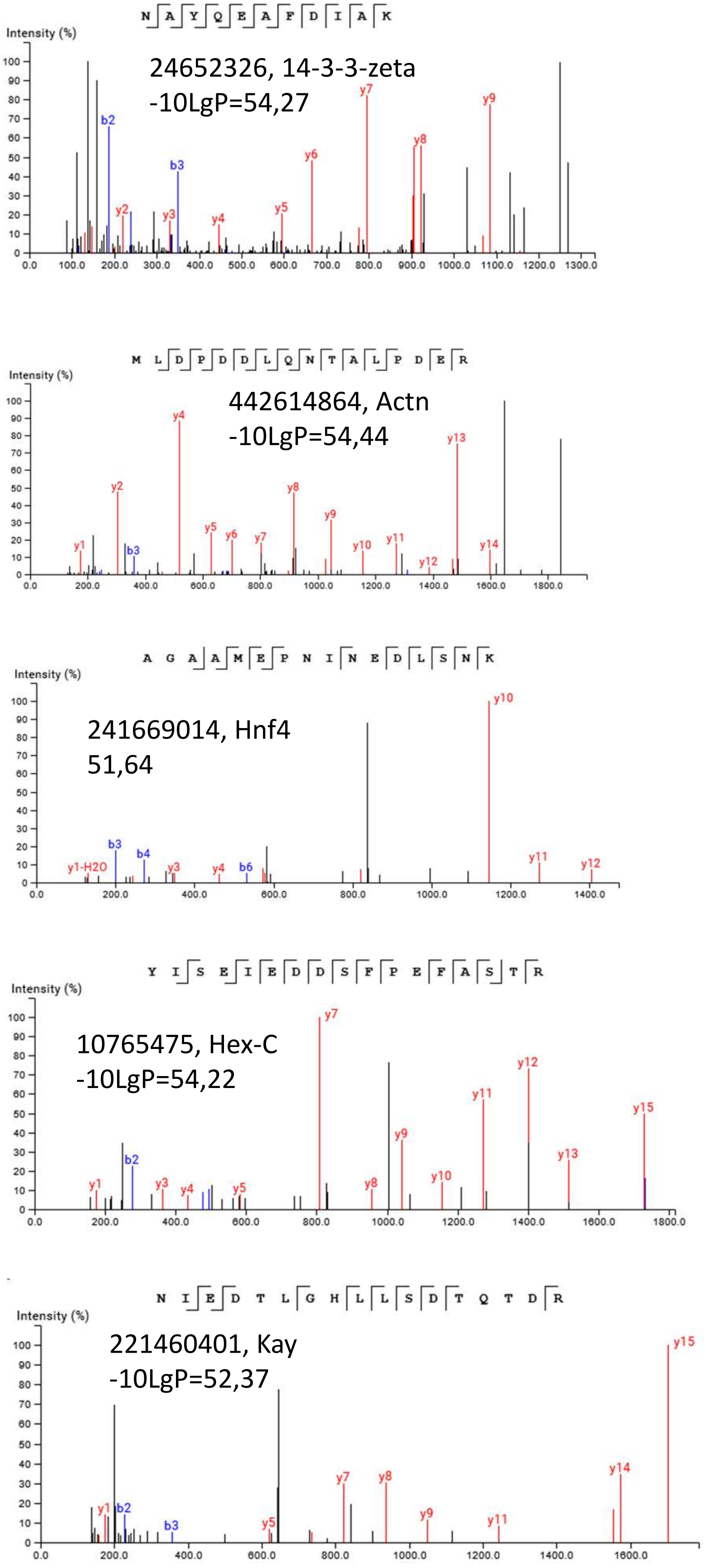
N A Y Q E A F D I A K
Intensity (%)
24652326, 14-3-3-zeta
-10LgP=54,27
M L D P D D L Q N T A L P D E R
Intensity (%)
442614864, Actn
-10LgP=54,44
A G A A M E P N I N E D L S N K
Intensity (%)
241669014, Hnf4
51,64
Y I S E I E D D S F P E F A S T R
Intensity (%)
10765475, Hex-C
-10LgP=54,22
N I E D T L G H L L S D T Q T D R
Intensity (%)
221460401, Kay
-10LgP=52,37

Figure S5

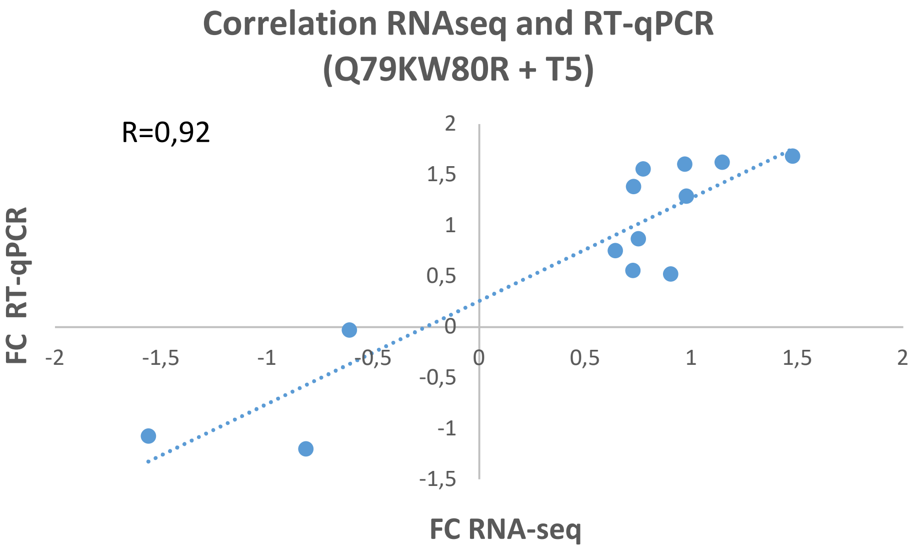

Correlation RNAseq and RT-qPCR
(Q79KW80R + T5)
R=0,92
FC RT-qPCR
FC RNA-seq
2
1,5
1
0,5
0
-0,5
-1
-1,5
-2

Figure S6

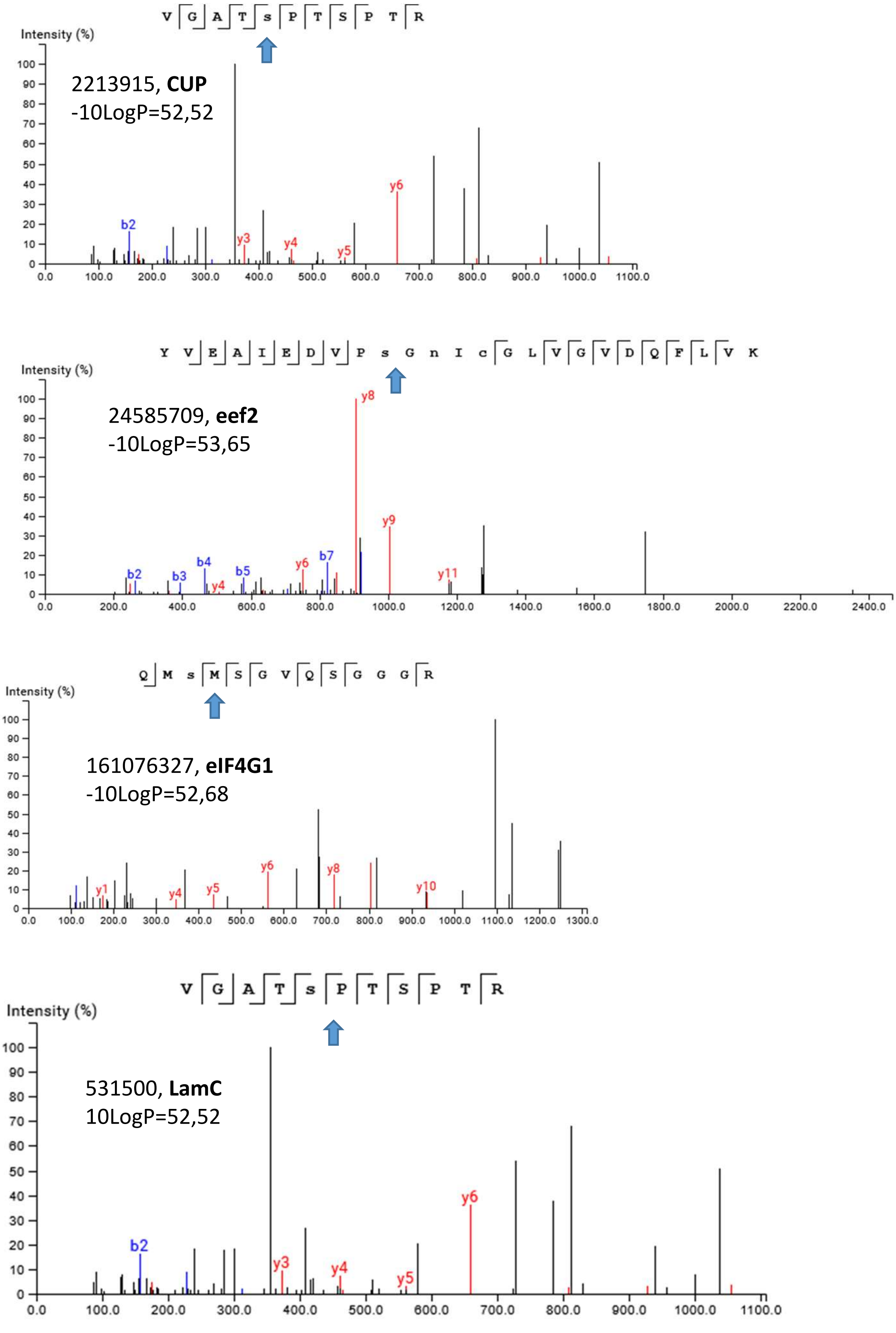
V G A T s P T S P T R
Intensity (%)
2213915, CUP
-10LogP=52,52
Y V E A I E D V P s G n I c G L V G V D Q F L V K
Intensity (%)
24585709, eef2
-10LogP=53,65
Q M s M S G V Q S G G G R
Intensity (%)
161076327, eIF4G1
-10LogP=52,68
V G A T s P T S P T R
Intensity (%)
531500, LamC
10LogP=52,52

Figure S7
